# Hepatocyte-specific S100a8 and S100a9 transgene expression in mice causes Cxcl1 induction and systemic neutrophil enrichment

**DOI:** 10.1186/1478-811X-10-40

**Published:** 2012-12-15

**Authors:** Lars Wiechert, Julia Németh, Tobias Pusterla, Christine Bauer, Aurora De Ponti, Sandra Manthey, Silke Marhenke, Arndt Vogel, Ursula Klingmüller, Jochen Hess, Peter Angel

**Affiliations:** 1 Division of Signal Transduction and Growth Control, DKFZ-ZMBH Alliance, German Cancer Research Center (DKFZ), Heidelberg, Germany; 2 Division of Systems Biology of Signal Transduction, DKFZ-ZMBH Alliance, German Cancer Research Center (DKFZ), Heidelberg, Germany; 3 Department of Hepatology, Medical School Hannover, Hannover, Germany; 4 Junior Group Molecular Mechanisms of Head and Neck Tumors, DKFZ-ZMBH Alliance, German Cancer Research Center (DKFZ), Heidelberg, Germany; 5 Department of Otolaryngology, Head and Neck Surgery, University Hospital Heidelberg, Heidelberg, Germany

**Keywords:** Calgranulins, Calprotectin, Hepatocytes, Neutrophils, Chemokines, Immune cell mobilization

## Abstract

**Background:**

Calprotectin consists of the Ca^2+^-binding proteins S100a8 and S100a9 that are induced in epithelial cells in response to tissue damage and infection. Both proteins are also secreted by activated innate immune cells and numerous studies demonstrate their crucial role in pathological conditions of acute and chronic inflammation.

**Results:**

Here, we established a conditional mouse model with simultaneous *S100a8* and *S100a9* transgene expression in hepatocytes (*TgS100a8a9*^*hep*^) under the control of doxycycline to unravel the role of epithelial-derived Calprotectin on tissue homeostasis and inflammation. *TgS100a8a9*^*hep*^ mice displayed a significant enrichment of neutrophils in peripheral blood and tissues with high blood content. Interestingly, *Cxcl1* transcription was significantly induced in the liver of *TgS100a8a9*^*hep*^ mice and primary hepatocytes derived thereof as compared to Control mice, accompanied by an increase of Cxcl1 serum levels. However, expression of other chemokines with a known function in neutrophil mobilization from the bone marrow, e.g. Csf3 and Cxcl2, was not altered. Doxycycline treatment of *TgS100a8a9*^*hep*^ mice reduced *Cxcl1* expression in the liver and resulted in normal numbers of neutrophils.

**Conclusion:**

In summary, our data demonstrate for the first time that hepatocyte-specific S100a8 and S100a9 expression induces a systemic mobilization of neutrophils by a specific activation of Cxcl1 transcription in the liver.

## Background

S100 proteins are a family of small Ca^2+^-binding proteins [1] which share a broad spectrum of functions including regulation of enzyme activity, Ca^2+^-homeostasis, and interaction with components of the cytoskeleton. Belonging to this family, S100A8 and S100A9 also known as migration inhibitory factor-related protein 8 (MRP8) and 14 (MRP14) are low molecular weight proteins of 8 kDa and 14 kDa, respectively, and were initially described as proteins expressed and released by infiltrating phagocytes [2,3]. S100A8 and S100A9 form preferably heterodimers also known as Calprotectin (S100A8/A9) whereas monomers and homodimers could not be detected in isolated granulocytes and monocytes [4]. Although expression of S100A8/A9 under physiologic conditions is restricted to cells of myeloid origin including circulating neutrophils, monocytes, and eosinophils [5,6], strong induction of S100A8/A9 was detected in epithelial and endothelial cells under inflammatory and stress conditions [7]. Furthermore, marked expression of S100A8/A9 was found in many tumors of epithelial origin including lung, breast, gastric, colorectal and prostate cancer [8]. These data strongly imply important functions of S100A8/A9 under conditions of tissue damage and inflammation. Accordingly S100A8/A9 is strongly induced in a broad variety of different chronic inflammatory disorders including rheumatoid arthritis, multiple sclerosis, inflammatory bowel disease, cystic fibrosis, arthritis, and psoriasis [8]. S100A8/A9 is able to support an inflammatory response via both intra- and extracellular mechanisms. In fact, intracellular S100A8/A9 has been shown to facilitate leukocyte trafficking through the transendothelial membrane via microtubule reorganization [9], upregulation of CD11b [10], as well as increase in the binding affinity of CD11b to the endothelial expressed integrin ICAM-1 [11]. Moreover S100A8/A9 is able to boost the inflammatory response by promotion of NADPH oxidase activity and production of ROS in phagocytes [12] and epithelial cells [13]. After secretion at the site of insult by epithelial and innate immune cells, S100A8/A9 directly acts as a chemoattractant for leukocytes, as demonstrated both *in vitro* and *in vivo*[14]. Importantly, S100A8/A9 is known to amplify pro-inflammatory signals, as it is able to induce the production and release of many cytokines and chemokines by epithelial and innate immune cells, e.g. CXCL1, CXCL2, IL-6, and TNF [15]. In this context S100A8/A9 was shown to promote pre-metastatic niche formation in the lungs of tumor-bearing mice [16,17]. Moreover, S100A8/A9 can promote its own expression by a feed-forward loop [15].

*In vivo* studies highlighted a crucial role for S100a8/a9 in the promotion of inflammation [18] and inflammation-induced carcinogenesis [19] by using *S100a9*^*-/-*^ animals in models of antigen-induced arthritis and colon carcinogenesis, respectively. However these studies focused on the role of myeloid-derived S100a8/a9. In contrast, no causal link between epithelial-derived S100a8/a9 and the establishment of chronic inflammation and subsequent tumor-development has been demonstrated yet. Strong experimental evidence indicates that S100A8/A9 overexpression in hepatocytes is associated with liver carcinogenesis as epidemiological studies showed a profound correlation of both proteins with high grade hepatocellular carcinoma in humans [20]. Moreover, our group demonstrated a strong increase in S100a8/a9 in the *Mdr2*^*-/-*^ model of inflammation-induced carcinogenesis in a NF-κB dependent manner [21]. Interestingly, elevated S100a8/a9 levels protected hepatocellular carcinoma cell lines from TNF-mediated apoptosis and promoted the production of ROS [21]. Moreover, liver injury induced by partial hepatectomy lead to induction of S100a8/a9 expression in hepatocytes, suggesting a role in liver regeneration [22]. Hitherto, we do not know whether the impact on pathological conditions of liver injury and carcinogenesis is mediated by well-known functions of S100a8/a9 on the amplification of pro-inflammatory signals, including immune cell recruitment and activation [15,23], because informative *in vivo* studies are still missing.

In contrast to other *in vivo* studies focusing on the function of heavily elevated systemic S100a8/a9 levels in settings of acute or chronic inflammation [18,24], we have established a conditional mouse model under the control of doxycycline to specifically investigate the effects of local S100a8/a9 overexpression without inducing a pathologic situation. In the present study we show that the simultaneous expression of S100a8 and S100a9 transgenes in hepatocytes leads to a constant homeostatic mobilization of neutrophils from the bone marrow via local induction of Cxcl1 expression. Importantly, the enrichment of the neutrophils occurs without activation and therefore hints to a so far unknown mechanism by which epithelial-derived S100a8/a9 may increase the surveillance capacity of an organism by innate immune cells.

## Results

### Conditional and hepatocyte-specific transgene expression in *TgS100a8a9*^*hep*^ mice

**Figure 1:**
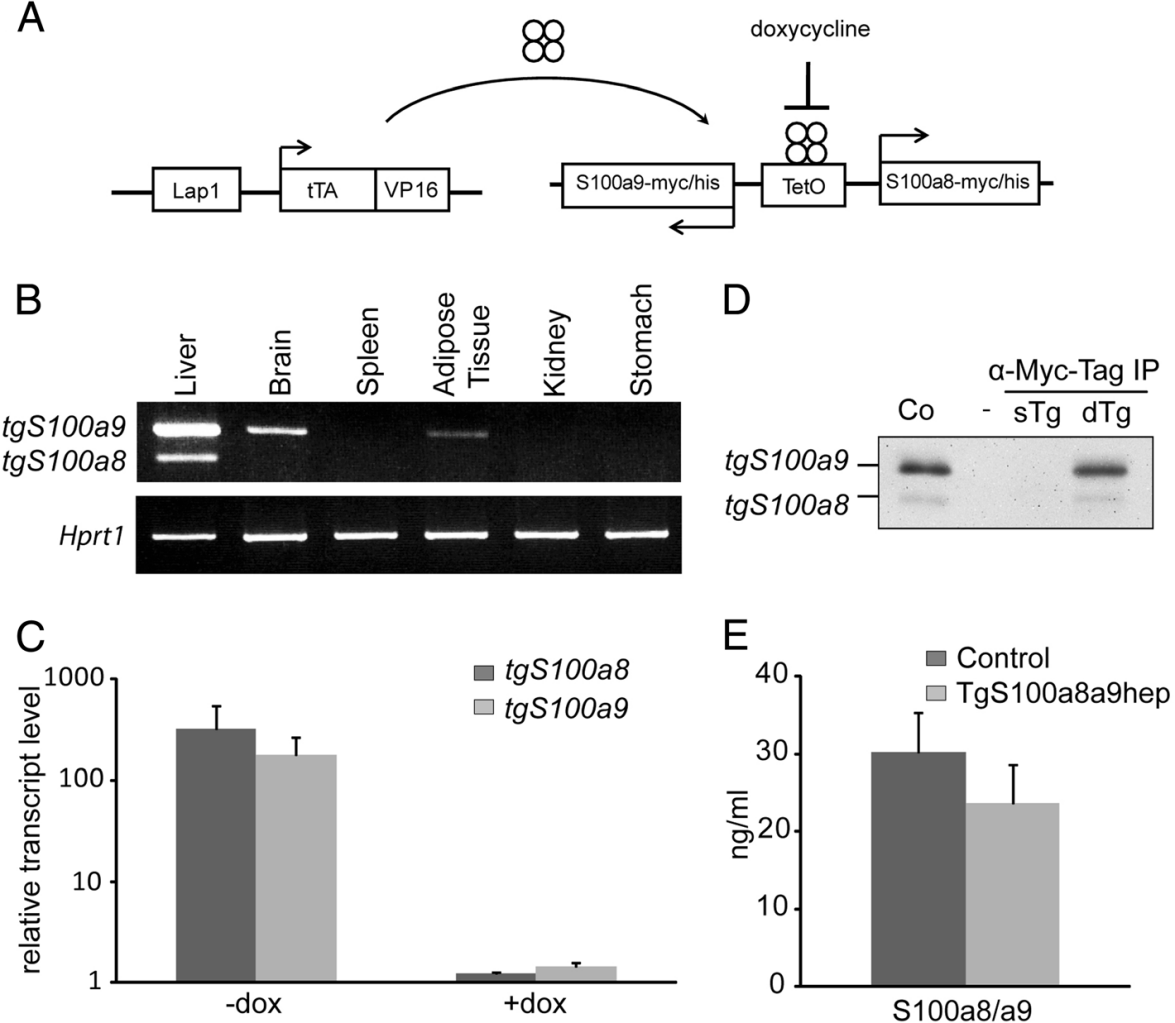
**Hepatocyte-specific and conditional S100a8 and S100a9 transgene expression in *TgS100a8a9***^***hep***^**mice.** (**A**) Schematic representation of the transgene construct: the transcriptional transactivator (tTA) is expressed under the control of the hepatocyte-specific promoter Lap1 (*TALap1*), binds to and activates the tetracycline operator (TetO), which drives the simultaneous expression of *S100a8-myc/his* and *S100a9-myc/his* transgenes (*TetOa8a9*). Doxycycline interferes with the binding of tTA to the TetO and inhibits transgene expression. (**B**) Tissue-specific tg*S100a8* and tg*S100a9* expression was determined by semi-quantitative PCR in different tissues from *TgS100a8a9*^*hep*^ mice using specific primers for transgenic *S100a8* and *S100a9* transcripts. *Hprt1* levels served as control for cDNA quantity and quality. (**C**) *TgS100a8a9*^*hep*^ mice were treated with (+dox) or without (-dox) doxycycline (10 μg/ml) for three weeks and transgene expression was assessed by qRT-PCR using liver cDNA. The mean value +SD of n=3 animals per group is depicted. (**D**) Whole liver lysates from a *TgS100a8a9*^*hep*^ mouse (dTg) and a single *TetOa8a9* transgenic (sTg) control were purified with an anti-Myc-tag IP Kit and expression of transgenic proteins was monitored by Western blot analysis using a His-tag specific antibody. HeLa cells were transiently transfected with *S100a8* and *S100a9* expression vectors and whole cell lysate was used as a positive control (Co). (**E**) S100a8/a9 protein was quantified by a heterodimer-specific ELISA in serum of Control (n=7) and *TgS100a8a9*^*hep*^ mice (n=6); mean +SD.

We established a new mouse line (*TgS100a8a9*^*hep*^) with conditional and hepatocyte-specific co-expression of S100a8 and S100a9 transgenes by using the Tetracyclin-inducible expression system (Tet-Off sytem) [25] to investigate the role of epithelial-derived S100a8/a9 under conditions of tissue homeostasis as well as pathological conditions. *TgS100a8a9*^*hep*^ mice were generated by breeding of the hepatocyte-specific transactivator (tTA) mouse line *TALap1* with the *TetOa8a9* transgenic mouse line, containing the Myc/His-tagged *S100a8* (*tgS100a8*) and *S100a9* (*tgS100a9*) transgenes under the control of a tTA-dependent and bidirectional promoter (Figure 1A). First, tissue-specific expression of *tgS100a8* and *tgS100a9* transcripts was confirmed by semi-quantitative PCR with cDNA from different tissues derived of *TgS100a8a9*^*hep*^ mice. Strong expression of both transgenes was detected in the liver, while minor *tgS100a9* transcript levels were also found in brain and adipose tissue, in line with published data on the *TALap1* transgene expression pattern (Figure 1B) [25]. In contrast, back skin, lung, heart, thymus, spleen, kidney, stomach, lymph nodes, and bone marrow displayed no expression for both transgenes (Figure 1B, Additional file 1: Figure S1A, data not shown). Quantification of *tgS100a8 and tgS100a9* transcripts in livers from six month-old *TgS100a8a9*^*hep*^ mice by qRT-PCR revealed a similar expression level compared to younger animals, demonstrating a persistent transgene expression over time (Additional file 1: Figure S1B). To confirm conditional transgene expression, *TgS100a8a9*^*hep*^ mice were treated with or without doxycycline for three weeks, and abolished transgene expression upon doxycycline treatment was measured by qRT-PCR as compared to control-treated mice (Figure 1C). Finally, expression of tgS100a8 and tgS100a9 proteins was found in *TgS100a8a9*^*hep*^ but not in Control mice by Western blot analysis, following enrichment from whole liver lysate (Figure 1D). However, immunohistochemical (IHC) staining of liver tissue sections did not show a strong signal for tgS100a8 and tgS100a9 proteins in hepatocytes of *TgS100a8a9*^*hep*^ mice, whereas S100a8 and S100a9 positive immune cells were easily detectable (data not shown), suggesting that only minor amounts of transgenic proteins were produced. In line with this data, an S100a8/a9 heterodimer-specific ELISA revealed comparable amounts of the S100a8/a9 protein complex in serum samples of peripheral blood from Control and *TgS100a8a9*^*hep*^ mice (Figure 1E). In summary, *TgS100a8a9*^*hep*^ mice were characterized by a conditional and tissue-specific transgene expression, which resulted in a low but persistent tgS100a8 and tgS100a9 protein level in the liver under conditions of normal tissue homeostasis.

### *TgS100a8a9*^*hep*^ mice develop a systemic enrichment in neutrophils

**Figure 2:**
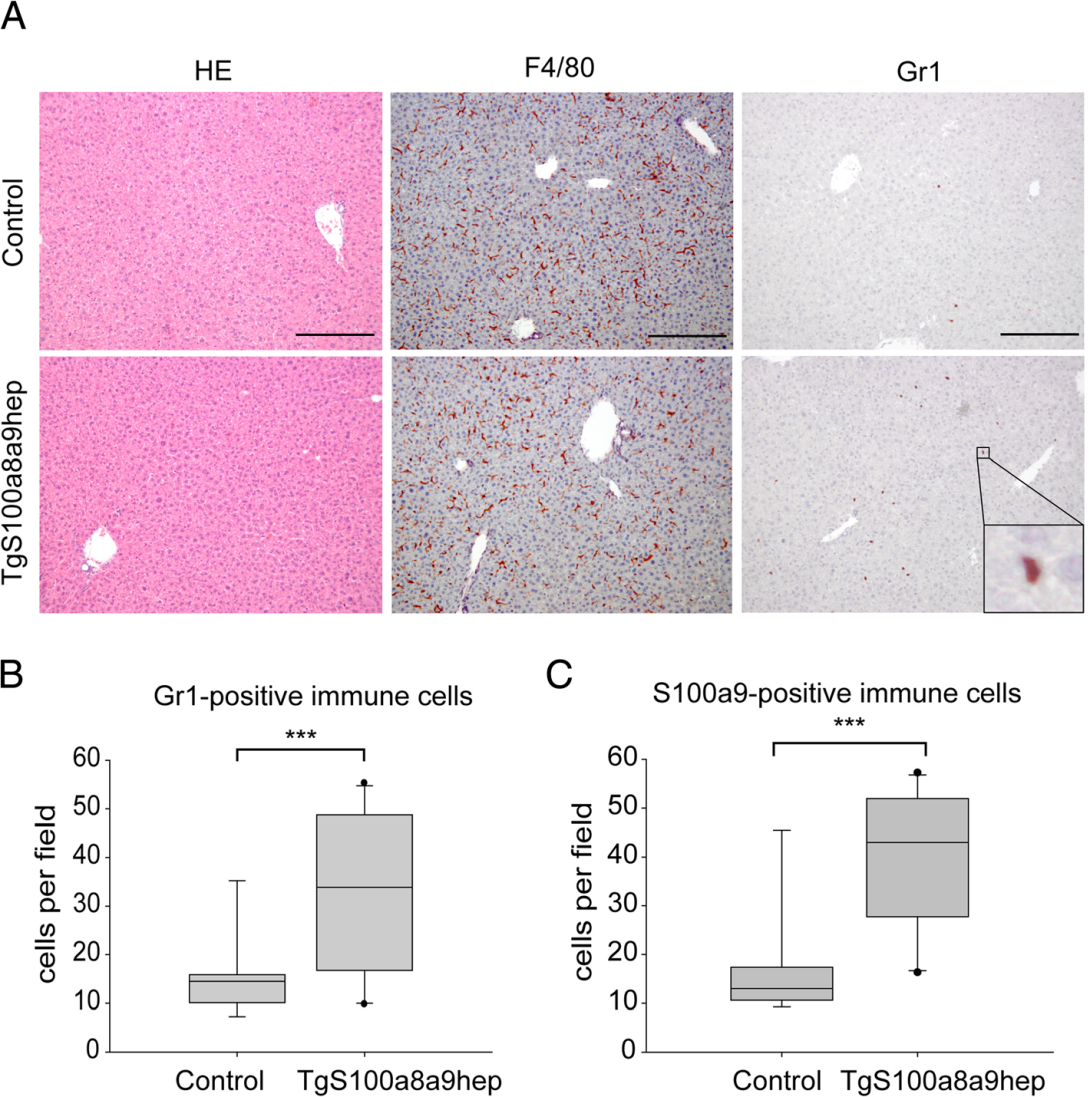
**Histological characterization of *TgS100a8a9***^***hep***^**liver sections.** (**A**) Tissue sections from Control and *TgS100a8a9*^*hep*^ mice were stained with hematoxylin and eosin (HE) or by IHC with specific antibodies for monocytes (F4/80) and granulocytes (Gr1). Representative images from at least n=5 mice are shown with red staining for F4/80 or Gr1, respectively, and counterstaining with hematoxylin. Bars represent 200 μm. Gr1-positive (**B**) and S100a9-positive (**C**) immune cells from five randomly taken pictures (magnification 50x) from Control (n=9) and *TgS100a8a9*^*hep*^ (n=10) were quantified. Box-plot shows median, 25 % and 75 % quartile (light grey box), 5 % and 95 % percentile (bars), students t-test, ***p≤0.001.

The S100a8/a9 protein complex is associated with inflammation [7] especially in the context of leukocyte chemoattraction and activation [14,26]. To investigate the impact of transgene expression on normal liver homeostasis, we analyzed tissue sections from eight week-old Control and *TgS100a8a9*^*hep*^ mice. Staining with hematoxylin and eosin (HE) revealed no obvious difference in liver architecture between *TgS100a8a9*^*hep*^ and Control littermates (Figure 2A). Lack of liver damage in response to tgS100a8 and tgS100a9 expression was further confirmed by similar AST and ALT levels in serum samples from Control and *TgS100a8a9*^*hep*^ mice (Additional file 2: Figure S2A, B). In addition, immunohistochemical staining with anti-HNE antibodies revealed no sign of increased ROS levels in *TgS100a8a9*^*hep*^ as compared to Control livers (Additional file 2: Figure S2C). Moreover, IHC staining for the monocyte-specific antigen F4/80 revealed a comparable number of Kupffer cells in both genotypes (Figure 2A). In contrast, the staining for Gr1, a marker specific for granulocytes as well as monocytes and myeloid derived suppressor cells (MDSCs), displayed a significant increase in Gr1-positive immune cells in *TgS100a8a9*^*hep*^ mice as compared to Control mice (Figure 2A and B).

IHC staining of liver sections with specific antibodies for S100a8 and S100a9, respectively, well-characterized markers for granulocytes and circulating monocytes [3,27], and subsequent quantification of S100a8 and S100a9 positive immune cells confirmed a significant increase of this subpopulation of immune cells (Figure 2C, data not shown). Indeed, comparison of the relative amount of Gr1- and S100a9-positive cells in the liver of Control and *TgS100a8a9*^*hep*^ mice revealed a high correlation coefficient (R^2^=0.95). Moreover, qRT-PCR analysis with cDNA of liver samples and primers recognizing selectively endogenous but not transgenic transcripts confirmed increased *S100a8* and *S100a9* expression in *TgS100a8a9*^*hep*^ compared to Control mice (Additional file 2: Figure S2D). In summary, our data supported a causal link between transgene expression and the accumulation of a distinct Gr1-positive immune cell subpopulation of the monocyte lineage in the liver of *TgS100a8a9*^*hep*^ mice.

To investigate whether the accumulation of this immune cell subpopulation increased with age or caused an alteration in liver architecture, liver sections of six month-old Control and *TgS100a8a9*^*hep*^ mice were stained with HE and for Gr1-positive cells by IHC staining. A substantial increase in Gr1-positive cells was still detectable in liver sections of six month-old *TgS100a8a9*^*hep*^ as compared to Control mice and no obvious difference was found when compared to younger *TgS100a8a9*^*hep*^ animals (Additional file 3: Figure S3).

**Figure 3:**
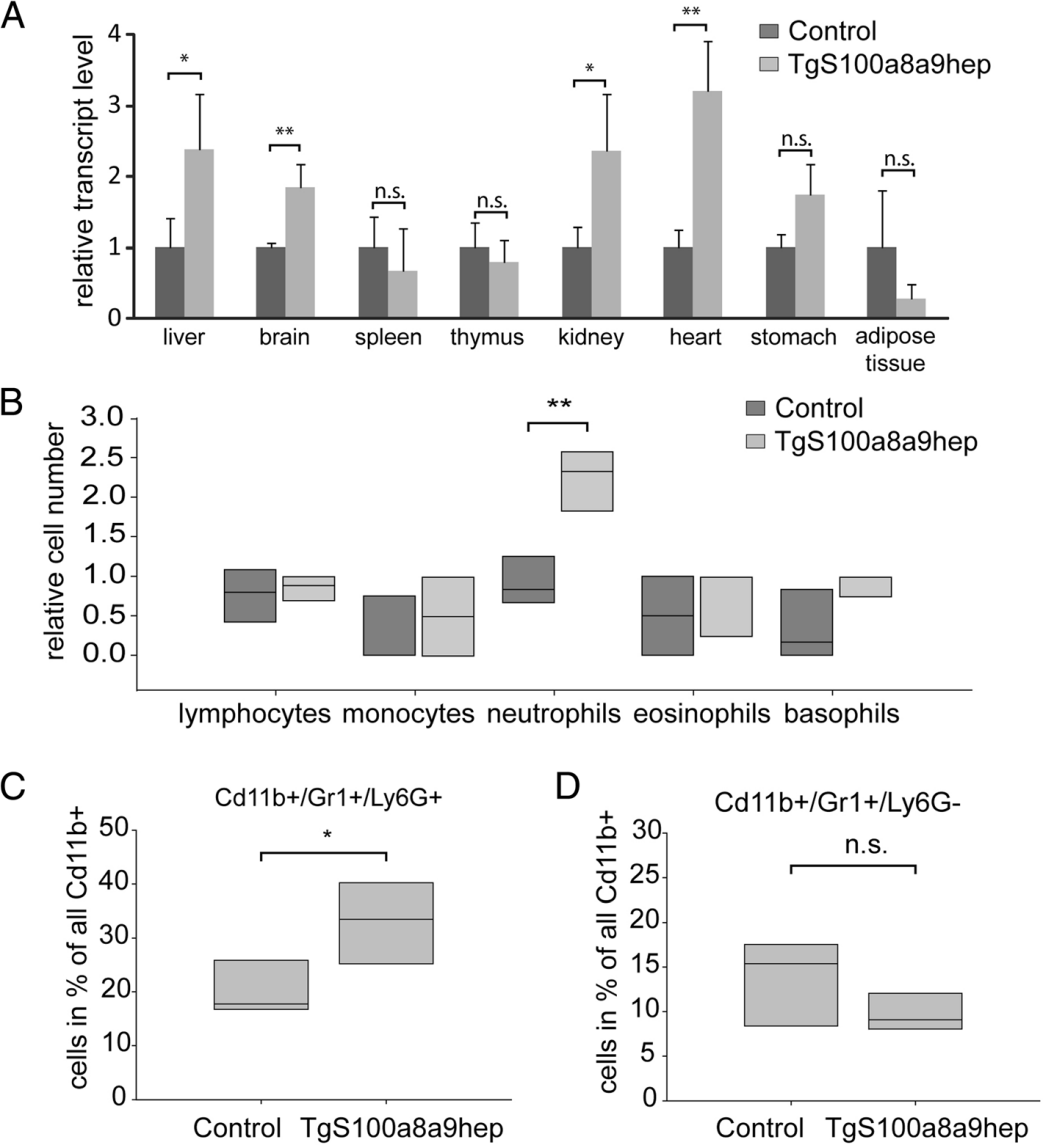
**Increased amount of neutrophils in peripheral tissue and blood samples.** (**A**) Relative expression of endogenous *S100a8* transcripts was determined by qRT-PCR using cDNA from different tissues of Control (n=3) and *TgS100a8a9*^*hep*^ (n=4) mice. Mean +SD, students t-test, *p≤0.05, **p≤0.01, n.s. not significant. (**B**) Leukocyte subpopulations in the peripheral blood from Control and *TgS100a8a9*^*hep*^ mice (n=4) were quantified and depicted as relative cell number. Box-plot shows median, 25 % and 75 % quartile (light and dark grey box), students t-test, **p≤0.01. (**C**-**D**) Peripheral blood from Control and *TgS100a8a9*^*hep*^ mice (n=7) was subjected to tri-color staining using antibodies against Cd11b (APC), Gr1 (PE), and Ly-6G (FITC). Graphs show the total percentage of Gr1+/Ly-6G+ cells (**C**) and Gr1+/Ly-6G- cells (**D**) from all Cd11b+ cells in peripheral blood. Box-plot shows median, 25 % and 75 % quartile (light grey box), students t-test, *p≤0.05, n.s. not significant.

It is worth mentioning that liver sections of *TgS100a8a9*^*hep*^ mice exhibited an equal distribution of Gr1-positive cells, suggesting that these cells were not actively recruited to the liver parenchyma but rather accumulated in the liver sinusoids due to increased amounts in the circulating blood. Accordingly, different tissues from Control and *TgS100a8a9*^*hep*^ mice were analyzed for the expression of endogenous *S100a8* and *S100a9* transcripts by qRT-PCR, which was used as surrogate marker for granulocytes. Elevated expression of endogenous *S100a8* transcript was detected in liver, brain, kidney, and heart from *TgS100a8a9*^*hep*^ (n=4) as compared to Control mice (n=3), whereas the expression of *S100a8* was not altered in spleen, thymus, stomach, and adipose tissue (Figure 3A). Similar results were measured for endogenous *S100a9* transcript levels by qRT-PCR (Additional file 4: Figure S4A), indicating a specific increase in the amount of S100a8/a9 positive immune cells in highly perfused tissues. In line with this assumption, an almost 2.5-fold enrichment of neutrophils, but not eosinophils, basophils, monocytes, or lymphocytes was found in peripheral blood counts of *TgS100a8a9*^*hep*^ as compared to Control mice (n=4, Figure 3B, Additional file 4: Figure S4B). Elevated levels of neutrophils were further confirmed by flow cytometric analysis using an antibody for Ly-6G, a specific marker for neutrophils [28,29], in combination with antibodies for Cd11b and Gr1 to distinguish the neutrophil subpopulation (Cd11b^+^/Gr1^+^/Ly-6G^+^) from other cell types (Cd11b^+^/Gr1^+^/Ly-6G^-^) including monocytes and monocytic MDSCs [28,30]. The total percentage of Gr1^+^/Ly-6G^+^ neutrophils within the Cd11b^+^ population was significantly increased in *TgS100a8a9*^*hep*^ as compared to Control mice (n=7, Figure 3C), whereas the relative number of the Gr1^+^/Ly-6G^-^ subpopulation was not affected by the transgene expression (Figure 3D). To exclude the possibility that Gr1-specific antibodies interfered with binding of anti-Ly-6G to its dedicated epitope, staining for neutrophils using an antibody specific for Ly-6C instead of anti-Gr1 was performed on wild type blood cells. Comparable amount of neutrophils were found with both staining procedures (Additional file 4: Figure S4C-E). In summary, our data demonstrate an age-independent systemic increase of neutrophils in the circulating blood of *TgS100a8a9*^*hep*^ mice, which resulted in the accumulation of these cells in peripheral tissues with a strong blood supply, but without obvious features of inflammation or signs of pathological condition.

### Systemically enriched neutrophils in *TgS100a8a9*^*hep*^ mice are not activated

**Figure 4:**
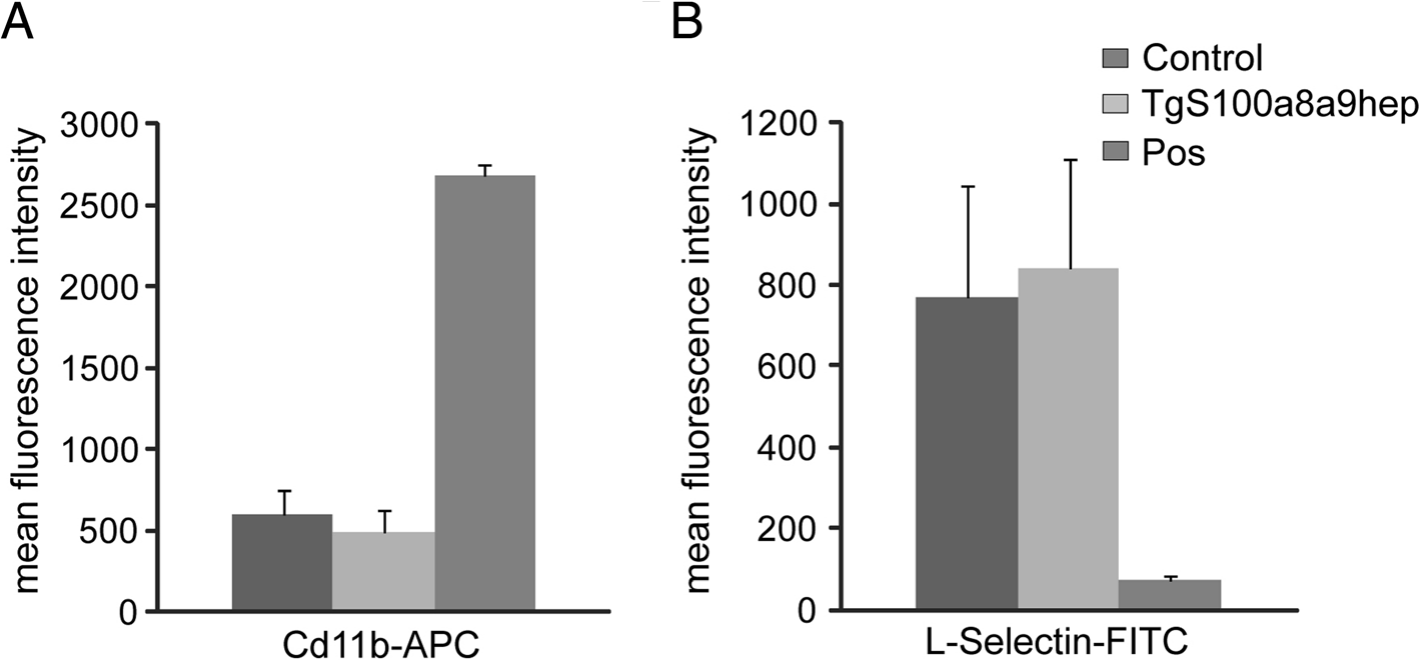
**Molecular markers of neutrophil activation in *TgS100a8a9***^***hep***^**mice.** Blood neutrophils (Gr1+/Ly-6G+ or Cd11b+/Gr1+) from Control and *TgS100a8a9*^*hep*^ mice or isolated neutrophils treated with 10 μg/ml TPA for 5 min (Pos) were stained with specific antibodies for (**A**) Cd11b (APC) and (**B**) L-Selectin (FITC), or the respective isotype control antibody. The mean fluorescence intensities from Control (Cd11b n=9; L-Selectin n=5), *TgS100a8a9*^*hep*^ (Cd11b n=8; L-Selectin n=5) and Pos (n=3) samples were determined and depicted as means +SEM.

Since *TgS100a8a9*^*hep*^ mice did not develop an obvious pathological phenotype with age, we investigated the activation state of the systemically enriched neutrophil subpopulation. Upregulation of Mac-1 (Cd11b/Cd18) and ectodomain shedding of L-Selectin (Cd62L), two frequently used activation markers for neutrophils [31], were determined by flow cytometry on peripheral blood neutrophils (Gr1^+^/Ly6G^+^ for Cd11b; Cd11b^+^/Ly6G^+^ for L-Selectin) derived from Control and *TgS100a8a9*^*hep*^ animals (n≥5). Importantly, no difference in the mean intensity was detectable for Cd11b and L-Selectin surface expression, while Cd11b upregulation and L-Selectin shedding was easily detectable upon TPA-stimulation of isolated neutrophils (Figure 4A, B and Additional file 5: Figure S5), suggesting an accelerated mobilization but no further activation of neutrophils in *TgS100a8a9*^*hep*^ mice.

### Altered expression of the chemokine Cxcl1 in the liver of *TgS100a8a9*^*hep*^ mice

**Figure 5:**
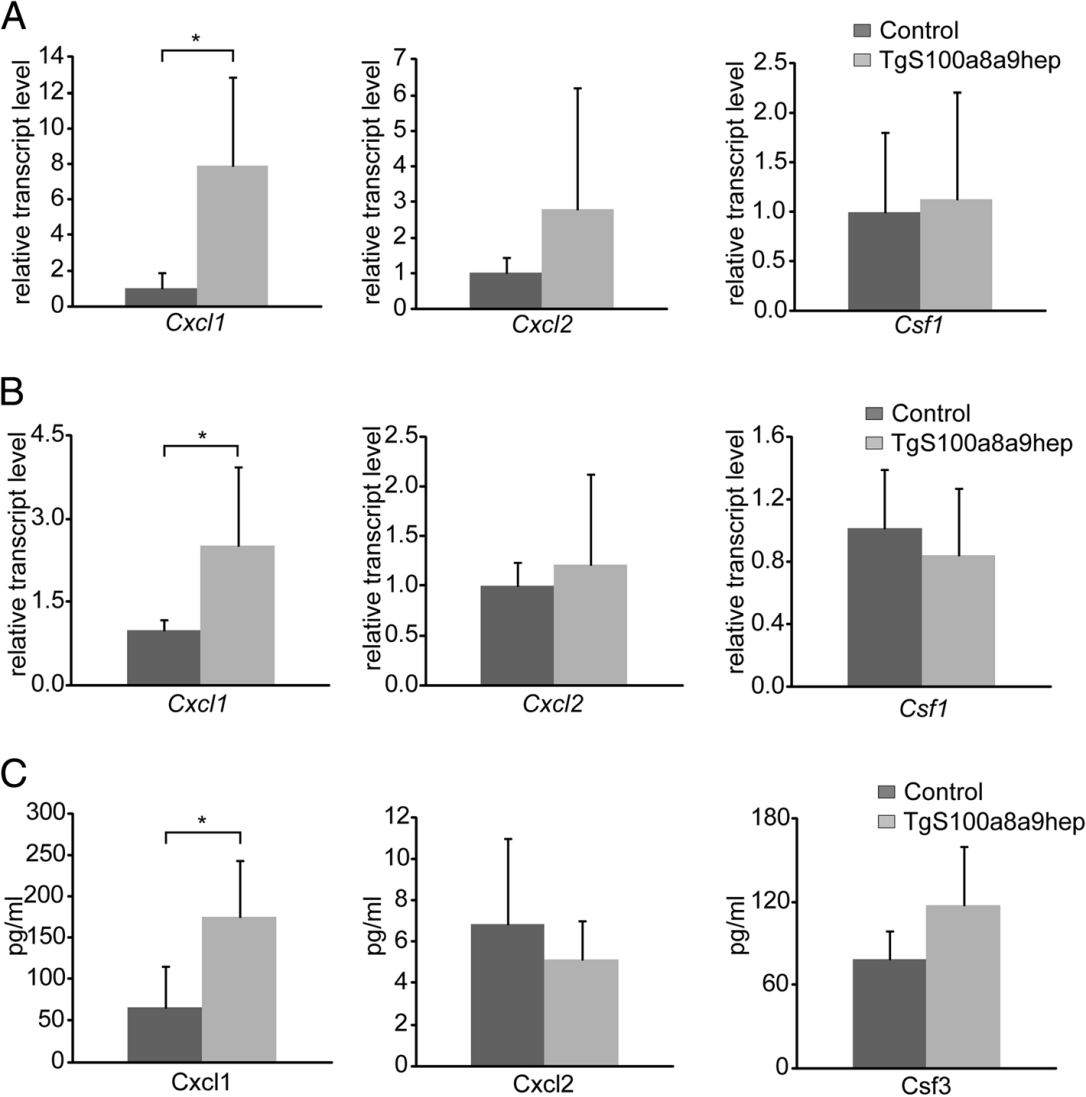
**Chemokine levels in liver, primary hepatocytes, and serum of Control and*TgS100a8a9***^***hep***^**mice.** (**A**) Relative expression of *Cxcl1*, *Cxcl2* and *Csf1* was determined by qRT-PCR analysis on liver cDNA from Control (n=6) and *TgS100a8a9*^*hep*^ (n=9) mice and is depicted as mean +SD. (**B**) Primary hepatocytes were isolated from Control and *TgS100a8a9*^*hep*^ mice and cultivated *in vitro* for 24 hours. Relative expression of *Cxcl1*, *Cxcl2*, *Csf1* was measured by qRT-PCR on cDNA from primary hepatocytes. Two biological replicates for each group were measured in triplicates and means are depicted +SD, students t-test, *p≤0.05. (**C**) Protein levels of Cxcl1, Cxcl2, and Csf3 in serum samples from Control and *TgS100a8a9*^*hep*^ (n≥4) mice were measured by ELISA. Mean +SD, students t-test, *p≤0.05.

As neutrophils have a relatively short life span in peripheral blood, continuous renewal by mobilization from the bone marrow is mandatory [32]. This homeostatic release from the bone marrow is tightly regulated by an equilibrium of retention and mobilization factors either keeping the neutrophils in the hematopoietic cords of the bone marrow or mediating the transition across the sinusoidal endothelium into the circulating blood [32]. Recent studies demonstrated the importance of several chemokines such as Cxcl1, Cxcl2, and Csf3 (G-Csf) and the interplay with their cognate receptors Cxcr2 and Cxcr4 on the surface of neutrophils in the regulation of homeostatic neutrophil mobilization [33,34]. Moreover, the factors Csf1 (M-Csf) and Csf2 (Gm-Csf) have been related to neutrophil mobilization under homeostasis as well [32]. Although, several reports showed S100a8/a9-mediated chemoattraction of neutrophils [14,16,24,26], a direct involvement of transgenic proteins in neutrophil mobilization in *TgS100a8a9*^*hep*^ mice was unlikely as no systemic difference in S100a8/a9 protein serum levels was detected (Figure 1E). Consequently, we hypothesized that local expression of transgenic S100a8 and S100a9 in the liver of *TgS100a8a9*^*hep*^ mice induced the expression of one or more factors, which in turn were able to mediate neutrophil mobilization from the bone marrow. First, we analyzed cDNA from livers of Control and *TgS100a8a9*^*hep*^ mice by qRT-PCR for the expression of *Csf1*, *Csf2, Csf3*, *Cxcl1*, and *Cxcl2*. Transcripts of *Csf2* and *Csf3* were not detectable (data not shown), whereas the expression of *Cxcl2* and *Csf1* revealed no significant difference between *TgS100a8a9*^*hep*^ and Control livers (Figure 5A). Interestingly, *Cxcl1* transcripts were significantly upregulated in *TgS100a8a9*^*hep*^ as compared to Control mice (Figure 5A).

To address the question whether hepatocytes are the source of increased *Cxcl1* transcript levels in *TgS100a8a9*^*hep*^ mice, livers of Control and *TgS100a8a9*^*hep*^ mice (n=3 each) were perfused with collagenase and the cell suspension was cultivated *in vitro* for 24 hours to obtain primary hepatocytes. One aliquot of the cell suspension was directly taken after enzymatic digestion and a significant increase in *Cxcl1* transcript levels in the liver of *TgS100a8a9*^*hep*^ mice was confirmed by qRT-PCR (Additional file 6: Figure S6A). Subsequent qRT-PCR analysis with cDNA from primary hepatocytes, which were cultured *in vitro* for 24 hours revealed a strong expression in *S100a8* and *S100a9* transgenes in *TgS100a8a9*^*hep*^ hepatocytes and a significant increase in the expression of *Cxcl1* in *TgS100a8a9*^*hep*^ as compared to Control hepatocytes, whereas *Cxcl2* and *Csf1* were not affected (Figure 5B and Additional file 6: Figure S6B). These data strongly suggest that hepatocytes are the main source of increased *Cxcl1* transcription in the liver of *TgS100a8a9*^*hep*^ mice.

Finally, we analyzed serum and liver samples of Control and *TgS100a8a9*^*hep*^ mice by enzyme-linked absorbent immunoassays (ELISA) for Cxcl1, Cxcl2, and Csf3 protein levels. Substantial levels of Cxcl1 and Csf3 were measured in both groups, whereas only low amounts were detected for Cxcl2 (Figure 5C). We found no major difference in Cxcl2 and Csf3 protein levels, but in accordance with the qRT-PCR data, Cxcl1 protein levels were significantly increased in *TgS100a8a9*^*hep*^ as compared to Control mice (Figure 5C and Additional file 7: Figure S7).

### Enhanced Cxcl1 expression and neutrophil enrichment in *TgS100a8a9*^*hep*^ mice depends on transgene expression

**Figure 6:**
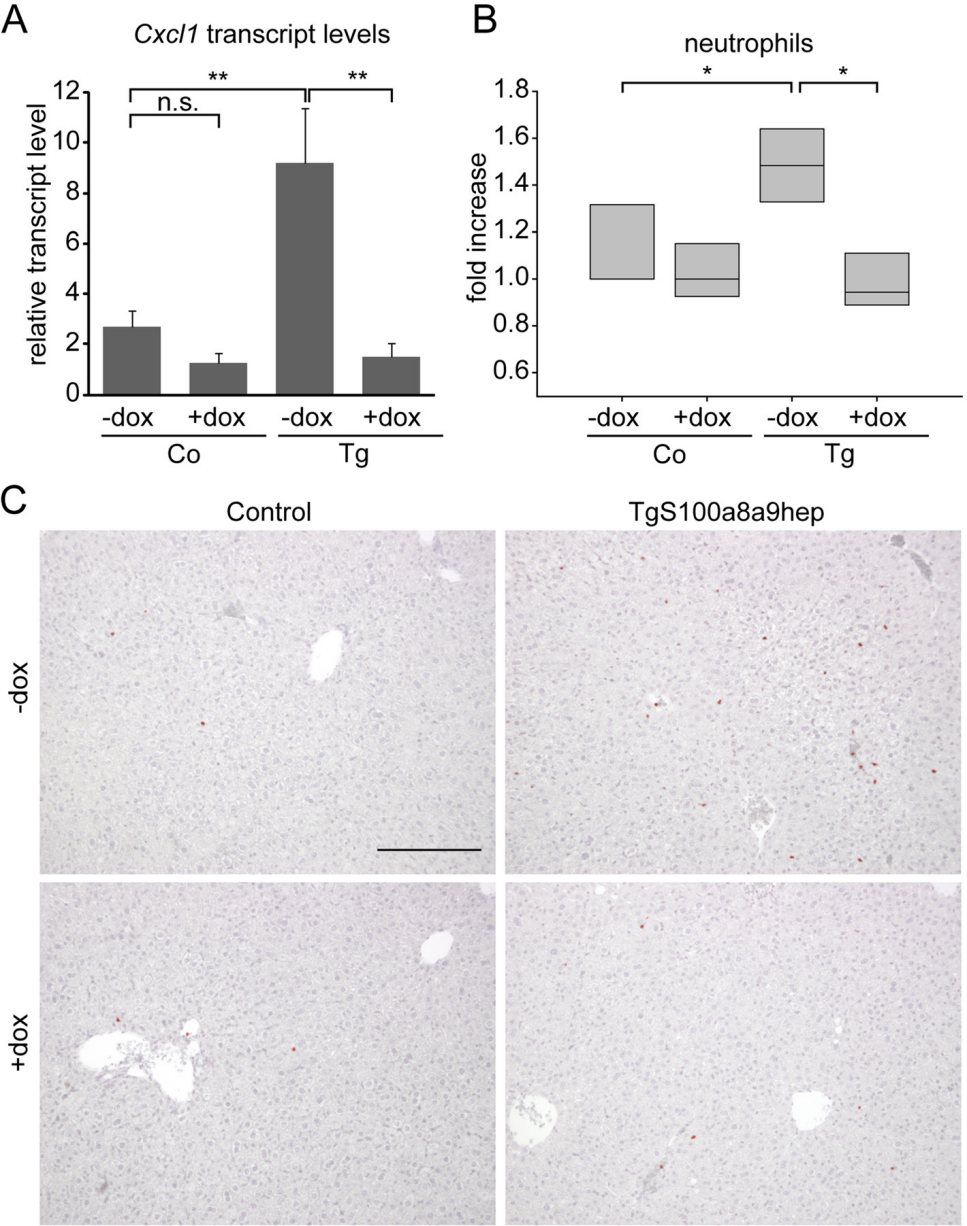
**Measurement of neutrophil numbers and Cxcl1 expression in Control and *TgS100a8a9***^***hep***^**mice treated with doxycycline.** Control (Co) and *TgS100a8a9*^*hep*^ (*Tg*) mice were treated with (Co +dox, *Tg* +dox) or without (Co -dox, *Tg* -dox) doxycycline (10 μg/ml) containing drinking water. (**A**) Relative expression of *Cxcl1* was determined by qRT-PCR on liver cDNA. Mean +SD, students t-test, *p≤0.05, **p≤0.01, n.s. not significant. (**B**) Peripheral blood cells were stained with specific antibodies for Cd11b (APC), Gr1 (PE) and Ly-6G (FITC) followed by flow cytometry analysis. Box-plot shows the relative fold increase of Gr1+/Ly-6G+ population from all Cd11b+ cells in peripheral blood; Median, 25% and 75% quartile (light grey boxes), students t-test, *p≤0.05. (**C**) IHC staining with Gr1-specific antibodies was performed on liver sections and representative images (n=4 animals per group) are shown with red staining for positively stained cells and counterstaining with hematoxilin. Bars represent 200 μm.

Control (Co) and *TgS100a8a9*^*hep*^ (*Tg*) animals were treated either with (*Tg* +dox, Co +dox) or without (*Tg* -dox, Co -dox) doxycycline-containing drinking water for three weeks to investigate, whether the observed increase in Cxcl1 levels and systemic enrichment of neutrophils critically depends on the presence of hepatocyte-specific tgS100a8 and tgS100a9 expression in adult mice. qRT-PCR analysis on livers from four animals per group, respectively, confirmed a significant increase in *Cxcl1* transcript levels in *Tg* -dox mice as compared to *Co* -dox animals. While doxycycline treatment had no impact on *Cxcl1* expression in *Co* +dox mice, the *Cxcl1* transcript levels were reduced to basal levels in *Tg* +dox mice upon doxycycline treatment (Figure 6A). In line with reduced Cxcl1 expression, we found a significant decreased in the amount of peripheral neutrophils in the blood of *Tg* +dox mice (Figure 6B, Additional file 8: Figure S8), as measured by FACS analysis and blood counts, accompanied by reduced neutrophil numbers in liver sections of *Tg* +dox as compared to *Tg* -dox littermates (Figure 6C). In contrast, doxycycline treatment did neither affect neutrophil numbers nor the number of Cd11b^+^/Gr1^+^/Ly6G^-^ cells in Co +dox mice (Figure 6, data not shown). Absolute numbers of neutrophils in blood counts from Control and *TgS100a8a9*^*hep*^ mice with and without doxycycline treatment revealed similar results (Additional file 8: Figure S8).

Taken together our data confirmed that the increase in Cxcl1 expression, which is accompanied by systemic enrichment of neutrophils in *TgS100a8a9*^*hep*^ mice critically depends on conditional S100a8 and S100a9 transgene expression in the liver.

### **The p38 MAPK pathway is inhibited in***TgS100a8a9*^*hep*^**hepatocytes and represents a negative regulator of Cxcl1 expression**

**Figure 7:**
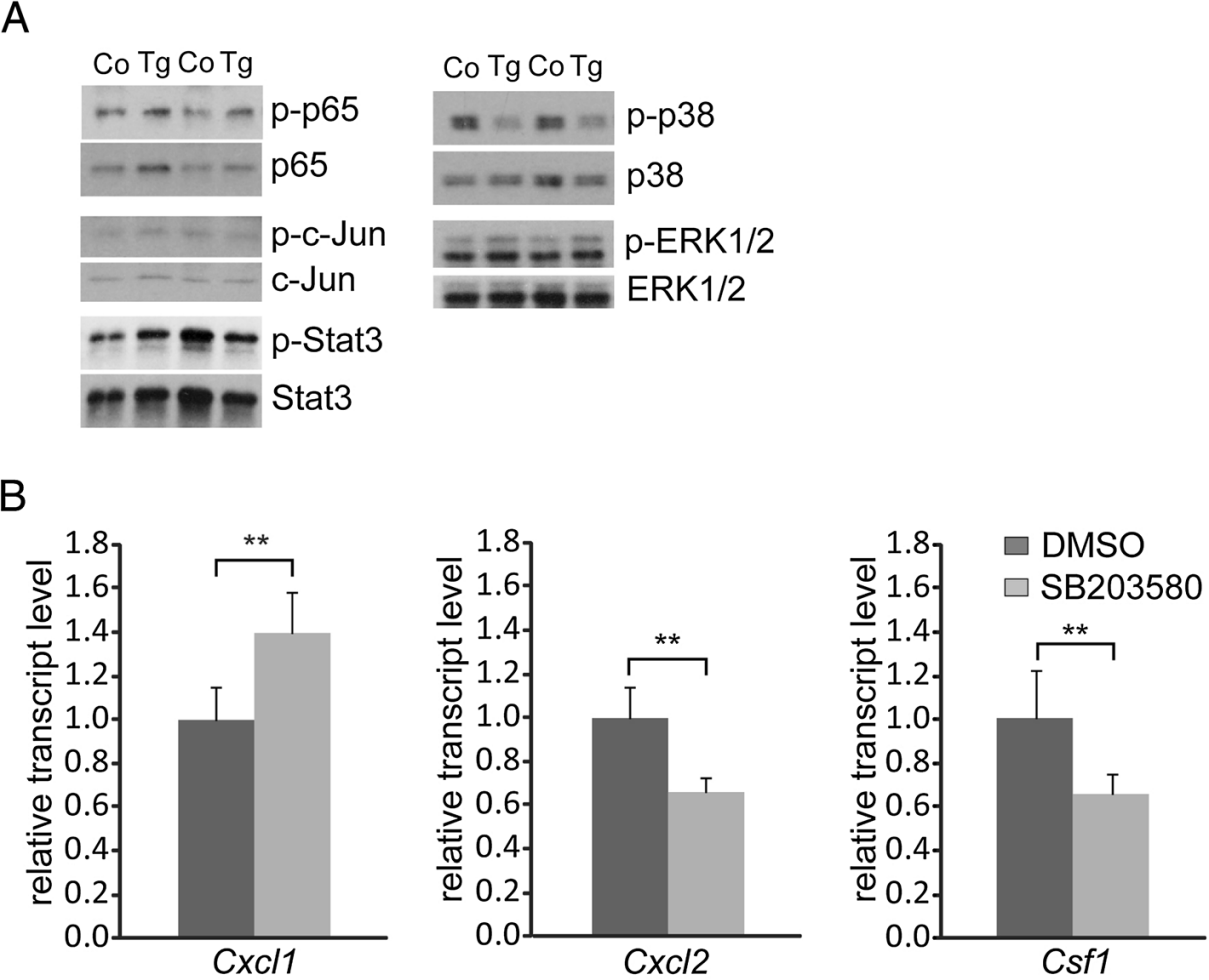
**Involvement of signaling pathways in *Cxcl1 *upregulation in hepatocytes.** (**A**) Primary hepatocytes were isolated from Control and *TgS100a8a9*^*hep*^ mice (n=2). Expression of p65, c-Jun, Stat3, p38, ERK1/2, and the corresponding phosphorylated forms was monitored by Western Blot analysis. (**B**) Primary hepatocytes were treated either with SB203580 or with (DMSO) for 5 h and relative expression of *Cxcl1*, *Cxcl2*, and *Csf1* was determined by qRT-PCR; n=5, Mean +SD, students t-test, **p≤0.01.

In order to unravel molecular mechanisms of transgene induce *Cxcl1* expression, we prepared protein lysate from freshly isolated primary hepatocytes derived from Control and *TgS100a8a9*^*hep*^ liver. Western Blot analysis revealed similar expression and phosphorylation of p65, c-Jun, Stat3, and ERK1/2, but a substantial decrease in phospho-p38 protein levels was evident in *TgS100a8a9*^*hep*^ as compared to Control hepatocytes (Figure 7A). Densitometric quantification of phosphorylated and total p38 protein levels confirmed a 50% reduced phosphorylation due to transgene expression (data not shown). To further confirm the role of phosphorylated p38 as a negative regulator for *Cxcl1* expression, freshly isolated wild type primary hepatocytes were treated with the p38 MAPK-specific inhibitor SB203580 for 5 hr and qRT-PCR analyses revealed a significant increase of *Cxcl1* transcript levels in hepatocytes with impaired p38 MAPK activity as compared to DMSO treated cells (Figure 7B). In contrast, *Cxcl2* and *Csf1* transcript levels were significantly decreased upon p38 inhibition, which is in line with our previous observation that tgS100a8/a9 expression specifically induces Cxcl1 expression in hepatocytes from *TgS100a8a9*^*hep*^ mice (Figure 5B). In summary, these data demonstrate that impaired p38 MAPK activity in the presence of tgS100a8/a9 expression triggers a specific induction of *Cxcl1* expression in hepatocytes.

## Discussion

A broad range of experimental studies demonstrate a direct link between S100a8/a9 protein expression and acute and chronic inflammation as well as inflammation-induced carcinogenesis [8]. In fact, S100a8/a9 is involved in several steps of the inflammatory response including leukocyte differentiation and chemoattraction, transendothelial migration, activation of immune and epithelial cells, and the induction of pro-inflammatory mediators [7]. Thereby S100a8/a9 might promote an inflammatory feed-forward loop, which facilitates the transition to a chronic inflammation. An established chronic inflammation strongly increases the likelihood of pathologic conditions such as tumor formation and is even considered as a hallmark of carcinogenesis [35]. Indeed *in vivo* studies with *S100a9*^*-/-*^ animals highlighted a crucial role for S100a8/a9 in the promotion of inflammation and inflammation-induced carcinogenesis in models of antigen-induced arthritis and colon carcinogenesis, respectively [18,19]. However these studies focused on the role of S100a8/a9 in the differentiation and function of myeloid-derived S100a8/a9. In contrast, no causal connection of epithelial-derived S100a8/a9 and the initiation and maintenance of a chronic inflammation and subsequent tumor-development have been demonstrated yet.

In order to investigate the role of epithelial-derived S100a8/a9 in liver homeostasis, we generated *TgS100a8a9*^*hep*^ mice with conditional and hepatocyte-specific S100a8 and S100a9 expression. The initial characterization revealed persistent transgene expression in liver. Furthermore the conditional *tgS100a8/a9* expression in *TgS100a8a9*^*hep*^ mice was confirmed via doxycycline treatment. Additionally, transgenic protein was detected in purified whole liver lysates from *TgS100a8a9*^*hep*^ mice, whereas the serum levels of total S100a8/a9 were unchanged in *TgS100a8a9*^*hep*^ mice compared to Controls. In summary the *TgS100a8a9*^*hep*^ mice showed conditional and hepatocyte-specific expression of *tgS100a8/a9* transcripts, which was not sufficient to reach a systemic increase of total S100a8/a9. Interestingly, all animal models, which were used so far to show S100a8/a9-dependent immune cell recruitment and activation accompanied by pathologic conditions were characterized by a strong S100a8/a9 overexpression [14,16,23,24]. In contrast, the use of the *TgS100a8a9*^*hep*^ mouse model enabled for the first time a detailed analysis of physiological and pathophysiological alterations in settings of low and locally restricted S100a8/a9 induction *in vivo*.

S100a8/a9 has been shown to act as a potent chemoattractant and activator for monocytes and neutrophils [14,21,23]. Therefore, tgS100a8/a9 expression might be expected to induce pathologic changes affecting liver homeostasis. However, *TgS100a8a9*^*hep*^ mice showed neither obvious differences in liver architecture and liver damage nor changes in the number of resident Kupffer cells. Interestingly, *TgS100a8a9*^*hep*^ animals developed a significant enrichment of Gr1-positive immune cells in the liver as compared to Control mice. Further characterization showed that this immune cell subpopulation was positive for endogenous S100a8 and S100a9 proteins and transcripts, and importantly, aged *TgS100a8a9*^*hep*^ animals exhibited a comparable enrichment of this specific immune subpopulation, supporting the existence of a constitutive signal in *TgS100a8a9*^*hep*^ mice, which leads to a sustained increase of these cells. Of note, the Gr1-positive subpopulation did not infiltrate into the liver parenchyma but rather accumulated in the blood and therefore appeared in the sinusoids of the liver. Therefore we hypothesized a systemic accumulation of Gr1-positive cells in *TgS100a8a9*^*hep*^ mice. Indeed, qRT-PCR analysis revealed an elevation of endogenous *S100a8/a9* transcripts, which was used as a surrogate marker for the affected subpopulation, also in other perfused tissues including brain, kidney, and heart. This further supported the hypothesis of a systemic enrichment in *TgS100a8a9*^*hep*^ mice, which was further confirmed by blood counts and flow cytometric analysis on peripheral blood samples. Although spleen is a highly perfused organ, it serves as marginating pool for granulocytes, ensuring rapid mobilization of innate immune cells upon infection and inflammation to facilitate an immediate inflammatory response [36]. Consequently, the overall amount of S100a8/a9 is very high and the difference due to the enriched population in *TgS100a8a9*^*hep*^ mice was not detectable.

Neutrophils circulating in the blood stream under healthy conditions, serve as first defense line against pathogens, and represent key players in sterile inflammatory diseases by secreting a plethora of different pro-inflammatory mediators [37]. Since the *TgS100a8a9*^*hep*^ mice developed no obvious pathological phenotype over time it was questioned whether the neutrophils were fully activated. Indeed, flow cytometric analysis on neutrophils of *TgS100a8a9*^*hep*^ mice revealed neither an increase in Cd11b expression nor ectodomain shedding of L-Selectin, which are well-known activation markers of neutrophils [31,38]. Together, these data convincingly show that the enriched neutrophils in the *TgS100a8a9*^*hep*^ mice are not activated, and additional signals are missing to induce an acute and/or chronic inflammation.

Injection of high amounts of S100a8/a9 was shown to directly act as a chemoattractant for neutrophils and lead to tissue-specific recruitment but was also reported as an activator of neutrophils [14,23]. Furthermore, a causal role for S100a8 in inducing myeloid cell migration has been demonstrated without further activation [39]. However *TgS100a8a9*^*hep*^ mice showed a systemic enrichment of neutrophils without activation and site-specific recruitment. This argues for a so far unknown mechanism of S100a8/a9, which might be only visible under conditions when tgS100a8/a9 expression is locally restricted to the liver without potent systemic accumulation. Hence we assumed that a mechanism, which is implicated in regulation of circulating neutrophils under homeostasis, was affected in *TgS100a8a9*^*hep*^ mice resulting in elevated neutrophil levels. The number of circulating neutrophils is mainly regulated via a constant release from the bone marrow compartment. This homeostatic release is realized either by Csf3, which is able to interfere with the Cxcl12-Cxcr4 mediated neutrophil retention [33,34,38], or by Cxcl1 and Cxcl2, which directly act as chemoattractants for neutrophils via Cxcr2 [40]. Moreover, experimental evidence suggests that other factors e.g. Csf1 and Csf2 might be involved in neutrophil mobilization under homeostasis [40,41]. Interestingly, only Cxcl1 was increased in the liver and serum of *TgS100a8a9*^*hep*^ compared to Control mice, whereas other factors were either not detectable or were not altered. Moreover, Cxcl1 was overexpressed in primary hepatocytes isolated from *TgS100a8a9*^*hep*^ mice, supporting that an increase in Cxcl1 expression is at least in part mediated by hepatocytes.

In line with our findings, recent reports demonstrated that administration of recombinant Cxcl1 in mice results in a three- to four-fold increase in the number of circulating neutrophils due to release from the bone marrow [34,42]. Accordingly, transgenic mice overexpressing *Cxcl1* in different tissues showed elevated numbers of circulating neutrophils [43]. It is worth mentioning that also Cxcl1-induced neutrophils showed no signs of further activation in terms of Cd11b upregulation and L-Selectin shedding [31]. On the other hand mice deficient for *Cxcr2* displayed an abnormal retention of neutrophils in the bone marrow whereas the numbers of circulating neutrophils largely declined [33].

**Figure 8:**
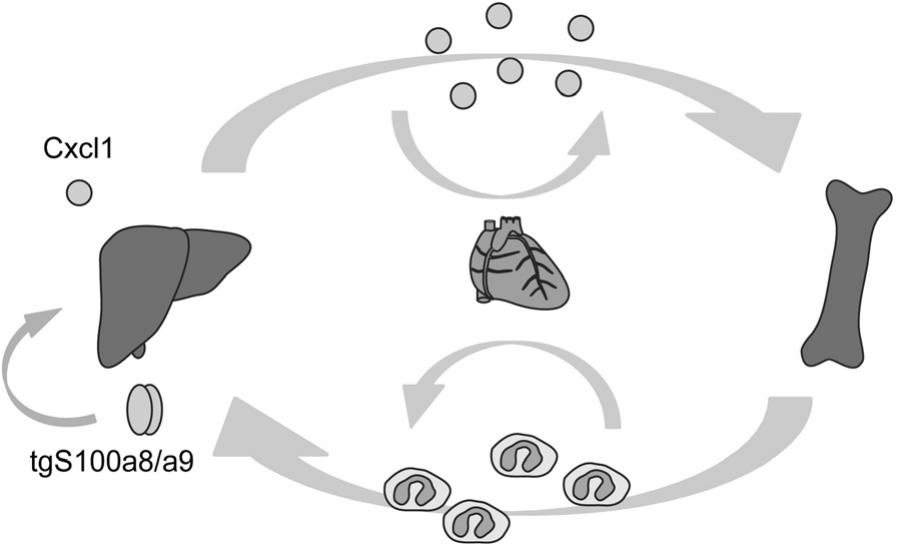
**Model of S100a8/a9-Cxcl1-mediated neutrophil enrichment in *TgS100a8a9***^***hep***^**mice.** The S100a8/a9 protein complex is produced in the liver of *TgS100a8a9*^*hep*^ mice and induces a specific expression of the neutrophil chemoattractant Cxcl1 in hepatocytes, which in turn leads to an increased pool of Cxcl1 protein in the serum of transgenic mice. Consequently, Cxcl1 enhances the release of neutrophils from the bone marrow and increases the number of circulating non-activated neutrophils, which also appear in distant organs such as liver.

To finally prove the causal link between transgene expression and the Cxcl1 induction as well as the accumulation of neutrophils in *TgS100a8a9*^*hep*^ mice, the transgene expression was repressed by doxycycline treatment. Indeed, *TgS100a8a9*^*hep*^ mice with repressed transgene expression exhibited baseline *Cxcl1* transcript levels in the liver, accompanied by a normal number of neutrophils in peripheral blood. These data supported the assumption that the expression of *Cxcl1* transcripts is causally linked to tgS100a8/a9 expression in the liver and that the accelerated mobilization of neutrophils from bone marrow is mediated by the specific increased of Cxcl1 expression in the liver of *TgS100a8a9*^*hep*^ mice (Figure 8).

The expression of *Cxcl1* is regulated by several transcription factors, including NF-κB [44], AP-1 [45], and Stat3 [46], which facilitate a rapid activation upon inflammatory stimulation. S100a8/a9 expressed by hepatocytes in *TgS100a8a9*^*hep*^ mice may be secreted in minor amounts and act in an autocrine manner via binding to Rage or Tlr4, both well established membrane receptors for extracellular S100a8/a9 [19,47]. Engagement of these receptors has been shown to induce relevant transcription factors for induced Cxcl1 transcription as well as their upstream signaling pathways, such as p38 and ERK1/2 MAPKs [48-50]. Western Blot analysis of primary hepatocytes revealed a substantial decrease in activation of the p38 MAPK pathway whereas other pathways were not altered. Inhibition of p38 activity was shown previously by our group and others in situations of intracellular S100a8/a9 expression [21,51]. In contrast, extracellular S100a8/a9 rather lead to a positive regulation of p38 [17,51]. Together with the finding that S100a8/a9 serum levels are unchanged in *TgS100a8a9*^*hep*^ as compared to Control and increased *Cxcl1* levels were observed in isolated hepatocytes our results point to an intracellular rather than an extracellular mode of action, how S100a8/a9 mediated *Cxcl1* induction. Finally, inhibition of p38 MAPK lead to increased expression of *Cxcl1* in primary hepatocytes by an as yet unknown mechanism indicating a role of p38 as a negative regulator for *Cxcl1* expression under conditions without inflammatory stimuli.

## Conclusion

*TgS100a8a9*^*hep*^ mice with conditional and hepatocyte-specific expression of S100a8 and S100a9 served as an useful tool to unravel a so far unknown function of calprotectin on the mobilization of neutrophils. Our new findings have a major impact on the current view, how calprotectin may influence acute and chronic inflammation under pathological conditions. Ichikawa et al. [19] demonstrated in a model of colitis-associated carcinogenesis that myeloid-derived S100a8/a9 induces chemokine expression by tumor cells, including Cxcl1, which was associated with an accumulation of Cd11b+/Gr1+ immune cells. However, *TgS100a8a9*^*hep*^ mice revealed a neutrophil mobilization upon expression of S100a8/a9 in non-transformed epithelial cells (Figure 8), and elucidated a complete new aspect of the role played by calprotectin in epithelial cells: S100a8/a9 may support an acute inflammatory response by increasing steady state levels of circulating neutrophils and thereby help to restore tissue homeostasis. However, under settings of sustained tissue damage the constitutive mobilization of neutrophils by a S100a8/a9-Cxcl1-dependent mechanism could enhance the risk for the development and maintenance of chronic inflammation.

## Methods

### Animals and animal work

Animals were maintained in a specific pathogen-free environment. All animal experiments were performed with age-matched male mice. The procedures for performing animal experiments were in accordance with the principles and guidelines of the Arbeitsgemeinschaft der Tierschutzbeauftragten in Baden-Württemberg and were approved by the German Regional Council in Karlsruhe.

*TALap1* mice were described previously [25]. In order to generate the *TetOa8a*9 mouse line, a bidirectional Tet-inducible expression plasmid was cloned from the parental pBi-L (Clontech Laboratories) encoding S100a8-myc/his and S100a9-myc/his fusion constructs driven by Tet-responsive promoter. The linearized pBi-S100a8a9 construct was injected into blastocytes of C57B1/6 mice. Genomic integration was determined by Southern Blot analysis.

For the analysis *TALap1* and *TetOa8a9* single transgenic mice served as Control animals. For doxycycline treatment, 6 to 8 week-old animals received 10 μg/ml doxycycline 5% sucrose containing drinking water *ad libitum* for a period of 3 weeks. Primary mouse hepatocytes were isolated and cultured as described [52]. Briefly, 8 week-old mice were anesthetized by intraperitoneal injection of 5 mg/100 mg body weight 10% ketamine hydrochloride and 1 mg/100 mg body weight 2% xylazine hydrochloride. After opening the abdominal cavity, the liver was perfused at 37°C with EGTA buffer (0.6% Glucose (w/v), 105 mM NaCl, 2.4 mM KCl, 1.2 mM KH_2_PO_4_, 26 mM Hepes, 490 μM L-Glutamine, 512 μM EGTA, 15% (v/v) amino acid solution, pH 8.3) via the portal vein for 5 min and subsequently for 5 min with Collagenase buffer (0.6% Glucose (w/v), 105 mM NaCl, 2.3 mM KCl, 1.2 mM KH_2_PO_4_, 25 mM Hepes, 490 μM L-Glutamine, 5.3 mM CaCl_2_, 12% (v/v) amino acid solution, 365 μg/ml collagenase type I-A, pH 8.3) until disintegration of the liver structure was observed. The liver capsule was removed, and the cell suspension was filtered through a 100 μm mesh. Cells were washed and, subsequently, viability of cells was determined by trypan blue staining. Two million living cells were seeded on collagen type I-coated 6 cm dishes and harvested after 24 hr. For *in vitro* inhibition of p38 MAPK, freshly isolated primary hepatocytes were treated with SB203580 (10 μM) or its solvent (DMSO) and harvested after 5 hr.

### Protein isolation and Western blot analysis

Preparation of whole cell extracts was done with radioimmunoprecipitation assay buffer (50 mM Tris-HCl pH 8.0, 150 mM NaCl, 0.1% SDS, 0.5% (v/v) sodium deoxylcholate, 1% (v/v) NP-40, proteinase inhibitor cocktail), and purification of Myc-tagged transgenic S100a8/a9 was performed with mammalian cMyc-tag/Co-IP Kit (Pierce) according to the manufacturer’s instructions. Proteins were separated on a 15% SDS-polyacrylamide gel in SDS-sample buffer, transferred to nitrocellulose membrane (Optitran BA-S83) and incubated with specific primary and HRP-labeled secondary antibodies (DakoCytomation). The blots were developed using the Classic E.O.S. developer (Agfa). Primary antibodies are listed in Additional file 9 (Table S1).

### Reagents

ELISAs for Cxc11, Cxcl2, G-Csf (all from R&D Systems), and S100A8/A9 (Immundiagnostik AG) were performed according to the manufacturer’s instructions.

### Immunohistochemistry and tissue staining

IHC analysis on paraffin sections was performed according to the manufacturer’s instructions (Vector Laboratories), and sections were counterstained with hematoxylin. Primary and secondary antibodies are listed in Additional file 9 (Table S1). Stained tissue sections were visualized and photographed using a Leica DMLB microscope and Olympus XC50 camera at a magnitude of 100x. Representative pictures for each genotype are shown.

### RNA preparation and quantitative real time polymerase chain reaction

Total RNA extraction was performed according to the manufacturer`s instructions using peqGOLD RNAPure (PeqLab Biotechnology). RNA was reverse transcribed using RevertAid H Minus-MuLV RT (Fermentas) and oligo-dT primers, followed by standard qRT-PCR performed on a detection system (StepOnePlus™; Applied Biosystems) using the Power SYBR® Green PCR Master Mix (Applied Biosystems). Quantitative real-time polymerase chain reaction (qRT-PCR) analysis was performed as described previously [53]. Primers used for qRT-PCR analysis are listed in Additional file 10 (Table S2). Target gene cycle of threshold values were normalized to the corresponding cycle of threshold values of *Hprt* using the change in cycle of threshold method.

### Preparation of blood samples and flow cytometry

For detection of immune subpopulations in peripheral blood, whole blood samples were mixed with EDTA (final 5 mM), subjected to erythrocyte lysis (ACK buffer, Lonza) and subsequently blockade of Fc-receptors (anti-Cd16/32, eBioscience). Staining was performed at 4°C for 30 min and cells were determined by flow cytometry analysis in FACSCalibur (BD Bioscience) using the software FlowJo (Tree Star, Inc.). Antibodies are listed in Additional file 9 (Table S1). As positive control for Cd11b upregulation and L-Selectin shedding, peripheral blood cells were treated with 10 μg/ml TPA for 5 min and stained as described.

### Digital images and statistical analysis

Digital images were elaborated using Photoshop CS3 (Adobe); the luminosity of brightest and dimmest pixel in each channel were adjusted to obtain the best visual reproduction, taking care to maintain linearity in the brightness scale. Error bars represent SD except were indicated. Statistical evaluation of obtained data was performed with SigmaPlot 11.0 (Systat Software Inc.).

## Abbreviations

ALT: Alanine transaminase; APC: Allophycocyanin; AST: Aspartate transaminase; CD: Cluster of differentiation; Csf: Colony-stimulatory factor; Cxcl: C-X-C motif ligand; Cxcr: C-X-C motif receptor; ELISA: Enzyme-linked absorbent immunoassay; FITC: Fluorescein isothiocyanate; G-Csf: Granulocyte colony-stimulatory factor; Gm-Csf: Granulocyte macrophage colony-stimulating factor; HE: Hematoxylin and eosin; Hprt: Hypoxanthine-guanine phosphoribosyltransferase; ICAM: Intercellular adhesion molecule; IHC: Immunohistochemistry; IL: Interleukin; Lap1: Liver activator protein 1; Ly6: Lymphocyte antigen 6; Mac-1: Macrophage-1 antigen; M-Csf: Macrophage colony-stimulating factor; Mdr2: Multidrug resistance 2; MDSCs: Myeloid derived suppressor cells; NADPH: Nicotinamid adenosine dinucleotide phosphate; PE: Phycoerythrin; qRT-PCR: Quantitative real-time PCR; ROS: Reactive oxygen species; tetO: Tetracyclin-operator; TgS100a8a9^hep^: Hepatocyte-specific S100a8/a9 transgenic mouse; TNF: Tumor necrosis factor; tTA: Tetracyclin transactivator.

## Competing interests

The authors declare that they have no competing interests.

## Authors’ contributions

LW, JN, TP, CB, AD, SMan, and SMar performed experiments; LW analyzed results, made figures, and wrote the paper; LW, AV, UK, JH, and PA designed the research. All authors read and approved the final version of this manuscript.

## Supplementary Material

Additional file 1 **Figure S1.***TgS100a8 and tgS100a9 expression in TgS100a8a9*^*hep*^*mice. Expression of tgS100a8 and tgS100a9* were analysed by qRT-PCR with **(A)** cDNA of different tissues (liver, lymph nodes, and bone marrow) from 8 weeks old *TgS100a8a9*^*hep*^ mice (n=3) or with **(B)** liver cDNA from two (n=5) and six (n=3) month old *TgS100a8a9*^*hep*^ mice; values were normalized to age-matched Control animals; mean, +SD. Click here for file

Additional file 2 **Figure S2.** Analysis of additional parameters of inflammation and liver damage in *TgS100a8a9*^*hep*^*mice. (A)* ALT and **(B)** AST serum levels in 8 weeks old *Control* and *TgS100a8a9*^*hep*^ mice (n=4). *Mdr2*^*-/-*^ mice served as positive control; mean, +SD. **(C)** Liver sections from Control and *TgS100a8a9*^*hep*^ mice were stained by IHC with specific antibodies for HNE adducts. Liver sections from *Mdr2*^*-/-*^ mice served as positive control for the staining. Representative images from at least n=3 mice are shown with red staining for HNE and counterstaining with hematoxylin. Bar represent 200 μm. (D) Relative levels of endogenous *S100a8* and *S100a9* transcripts was measured by qRT-PCR with liver cDNA from Control (n=9) and *TgS100a8a9*^*hep*^ (n=10) mice; mean, +SD, students t-test, *p≤0.05. Click here for file

Additional file 3 **Figure S3.** Histological and immunohistochemical characterization of six month old *TgS100a8a9*^*hep*^*mice.* Liver sections from six month old Control and *TgS100a8a9*^*hep*^ mice were stained with hematoxylin and eosin (HE) or by IHC with a specific antibody for granulocytes (Gr1). Representative images are shown with red staining for Gr1 (n=2 mice per group), and counterstaining with hematoxylin. Bars represent 200 μm. Click here for file

Additional file 4 **Figure S4.** Systemic enrichment of neutrophils in *TgS100a8a9*^*hep*^*mice. (A)* Relative expression of endogenous *S100a9* transcripts was determined by qRT-PCR using cDNA from different tissues of Control (n=3) and *TgS100a8a9*^*hep*^ (n=4) mice. Mean +SD, students t-test, *p≤0.05, **p≤0.01, n.s. not significant. **(B)** Absolute number of neutrophils in peripheral blood from Control and *TgS100a8a9*^*hep*^ mice (n=4) as measured by means of blood counts. **(C-E)** Peripheral blood from Control mice (n=8) was subjected to tri-color staining using antibodies against Cd11b (APC), Ly-6G (FITC), and Gr1 (PE) or Ly-6C (PE), respectively. Representative images of either **(C)** Gr1/Ly-6G or **(D)** Ly-6C/Ly-6G populations are shown. **(E)** Graph shows the total percentage of Gr1+/Ly-6G+ cells and Ly-6C+/Ly-6G+ cells from all Cd11b+ cells in peripheral blood; +SD. Click here for file

Additional file 5 **Figure S5.** Molecular markers of neutrophil activation in *TgS100a8a9*^*hep*^*mice.* Blood neutrophils (Gr1+/Ly6G+ or Cd11b+/Ly6G+) from Control and *TgS100a8a9*^*hep*^ mice were stained with specific antibodies for Cd11b (APC) and L-Selectin (PE), or the respective isotype control antibody. Representative histograms of Cd11b **(A)** and L-Selectin **(B)** stainings are shown for Control (red) and *TgS100a8a9*^*hep*^ (green) mice, including the staining with their respective isotype control (black). Click here for file

Additional file 6 **Figure S6.***Cxcl1 and tgS100a8/a9 expression in primary hepatocytes from Control and TgS100a8a9*^*hep*^*mice. (A)* Relative *Cxcl1* transcript levels was determined by qRT-PCR analysis with cDNA from cell suspension derived from collagenase perfused livers of Control and *TgS100a8a9*^*hep*^ mice. **(B)** Primary hepatocytes were cultivated *in vitro* for 24 hours and relative levels of *tgS100a8* and *tgS100a9* transcripts were measured by qRT-PCR. Two biological replicates for each group were measured in triplicates and means are depicted +SD, students t-test, **p≤0.01. Click here for file

Additional file 7 **Figure S7.** Cxcl1 protein expression in liver lysates from Control and *TgS100a8a9*^*hep*^*mice.* Protein levels of Cxcl1 in liver lysate from Control and *TgS100a8a9*^*hep*^ mice (n=4) were measured by ELISA. Mean +SD, students t-test, **p≤0.01. Click here for file

Additional file 8 **Figure S8.** Peripheral neutrophils in Control and *TgS100a8a9*^*hep*^*mice treated with doxycycline.* Control (Co) and *TgS100a8a9*^*hep*^ (*Tg*) mice were treated with (Co +dox, *Tg* +dox) or without (Co -dox, *Tg* -dox) doxycycline (10 μg/ml) containing drinking water. Peripheral blood neutrophils were measured by means of blood counts and depicted as absolute numbers. Box-plot shows median, 25 % and 75 % quartile (light grey box). Click here for file

Additional file 9 **Table S1.** Antibodies used for IHC, Western blotting, and flow cytometry. Click here for file

Additional file 10 **Table S2.** Primer pairs used for qRT-PCR analysis. Click here for file
