# Supplementary figures and images for: Hepatocyte-specific S100a8 and S100a9 transgene expression in mice causes Cxcl1 induction and systemic neutrophil enrichment
Source: Cell Commun Signal. 2012 Dec 15;10:40. doi: 10.1186/1478-811X-10-40 (PMC3533587; doi:10.1186/1478-811X-10-40)

**A**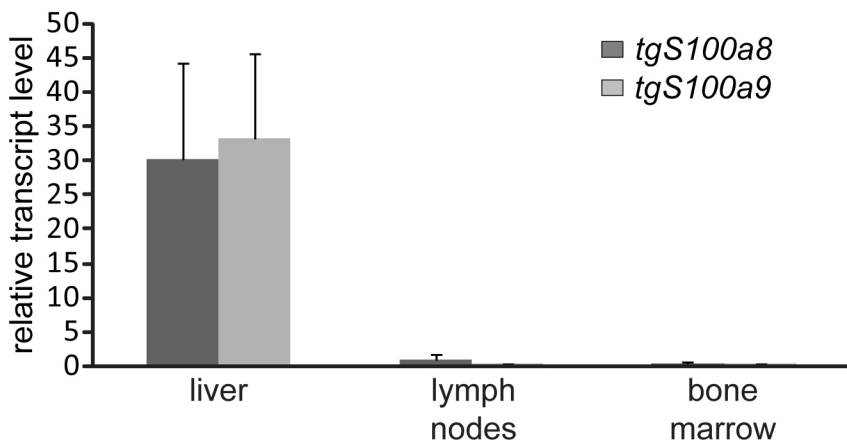**B**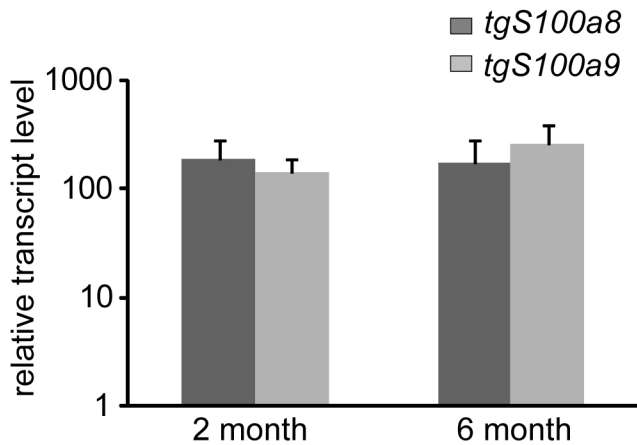

Supplement: Additional file 1 — Figure S1.TgS100a8 and tgS100a9 expression in TgS100a8a9hepmice. Expression of tgS100a8 and tgS100a9 were analysed by qRT-PCR with (A) cDNA of different tissues (liver, lymph nodes, and bone marrow) from 8 weeks old TgS100a8a9hep mice (n=3) or with (B) liver cDNA from two (n=5) and six (n=3) month old TgS100a8a9hep mice; values were normalized to age-matched Control animals; mean, +SD. [file 1478-811X-10-40-S1.pdf]

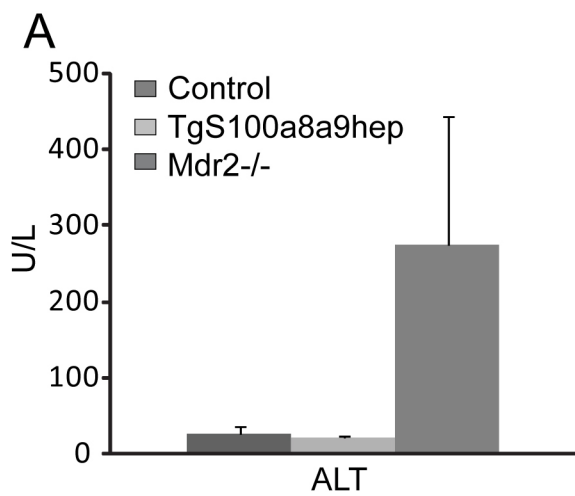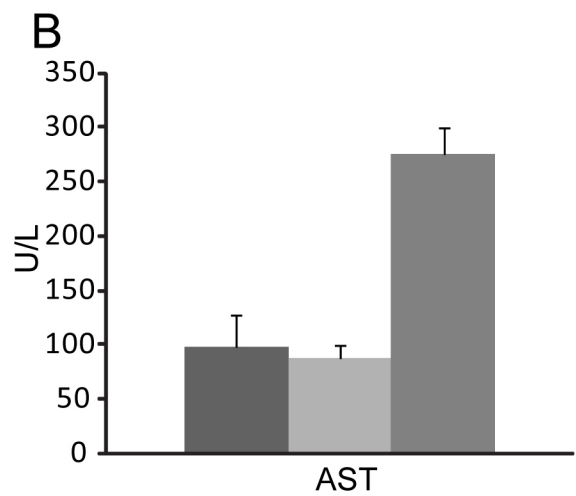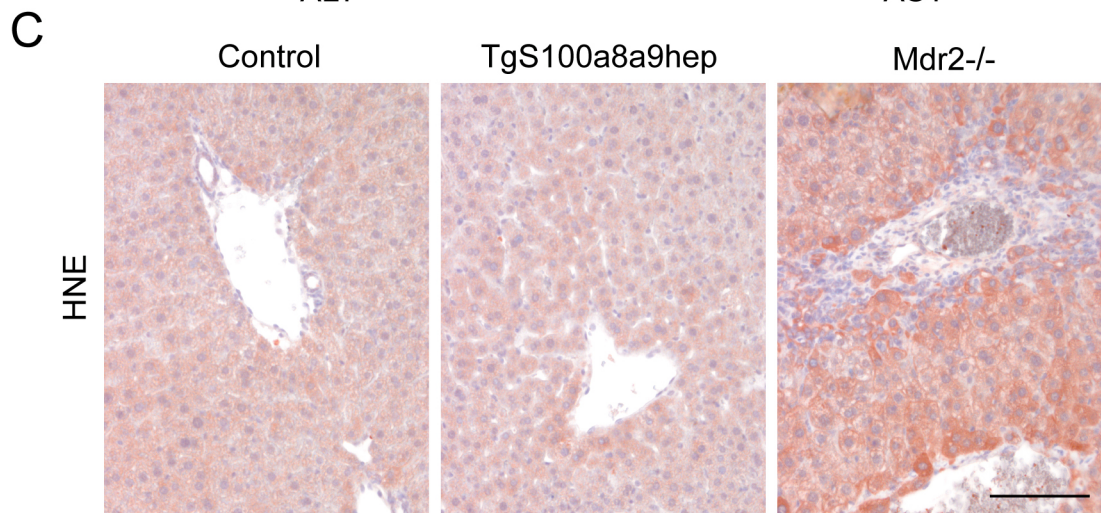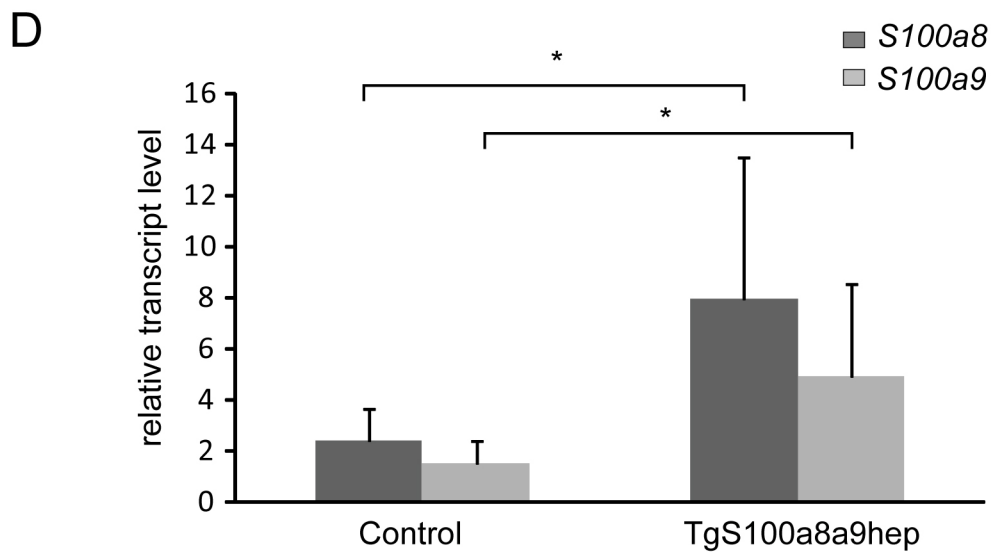

Supplement: Additional file 2 — Figure S2. Analysis of additional parameters of inflammation and liver damage in TgS100a8a9hepmice. (A) ALT and (B) AST serum levels in 8 weeks old Control and TgS100a8a9hep mice (n=4). Mdr2-/- mice served as positive control; mean, +SD. (C) Liver sections from Control and TgS100a8a9hep mice were stained by IHC with specific antibodies for HNE adducts. Liver sections from Mdr2-/- mice served as positive control for the staining. Representative images from at least n=3 mice are shown with red staining for HNE and counterstaining with hematoxylin. Bar represent 200 μm. (D) Relative levels of endogenous S100a8 and S100a9 transcripts was measured by qRT-PCR with liver cDNA from Control (n=9) and TgS100a8a9hep (n=10) mice; mean, +SD, students t-test, *p≤0.05. [file 1478-811X-10-40-S2.pdf]

Control

TgS100a8a9hep

HE

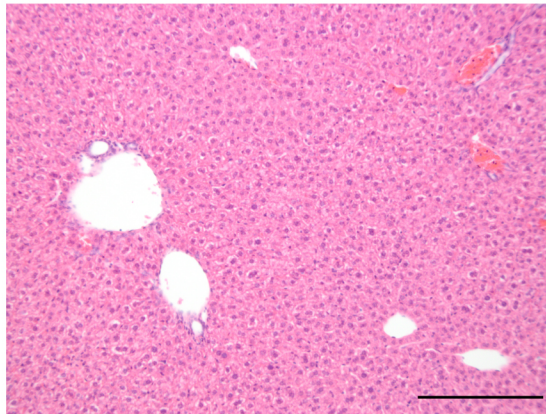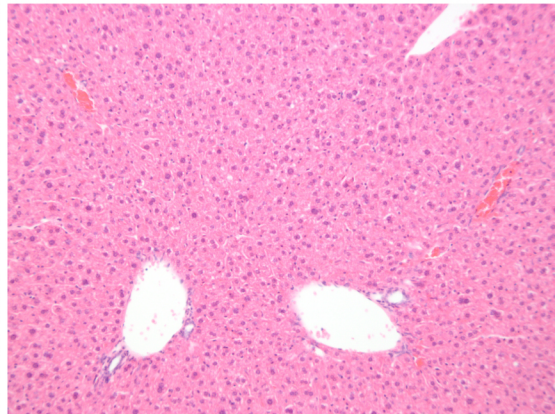

Gr1

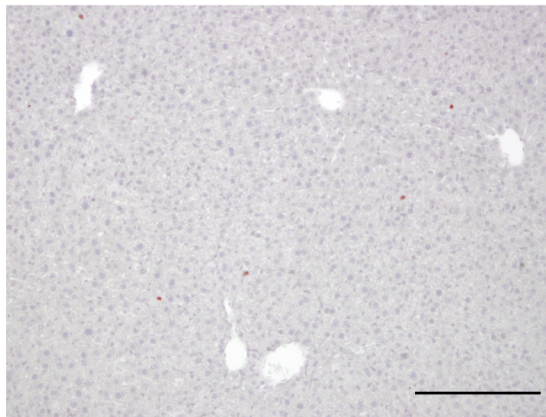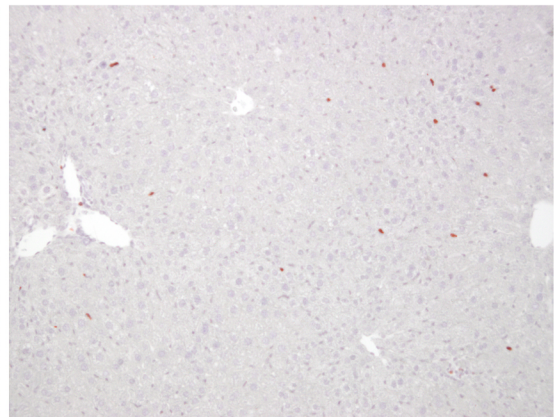

Supplement: Additional file 3 — Figure S3. Histological and immunohistochemical characterization of six month old TgS100a8a9hepmice. Liver sections from six month old Control and TgS100a8a9hep mice were stained with hematoxylin and eosin (HE) or by IHC with a specific antibody for granulocytes (Gr1). Representative images are shown with red staining for Gr1 (n=2 mice per group), and counterstaining with hematoxylin. Bars represent 200 μm. [file 1478-811X-10-40-S3.pdf]

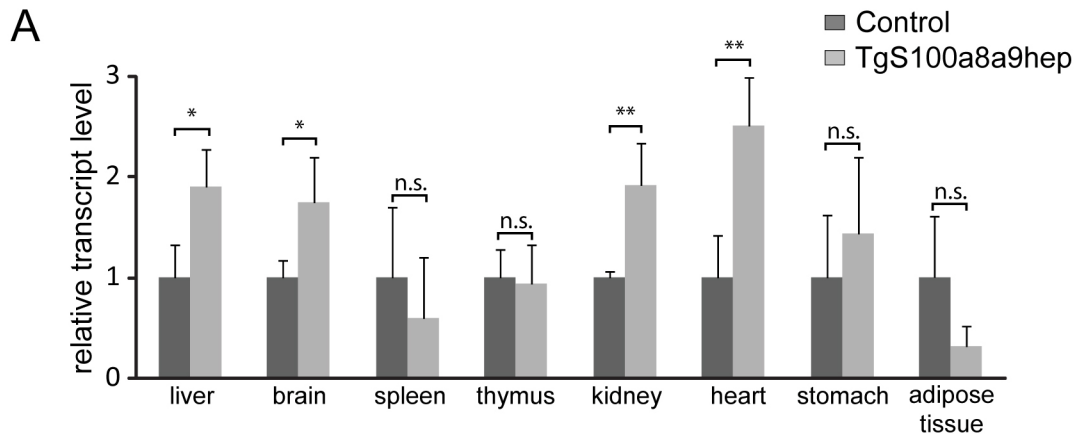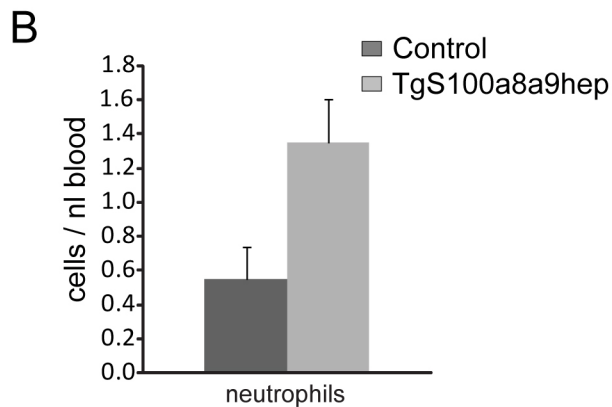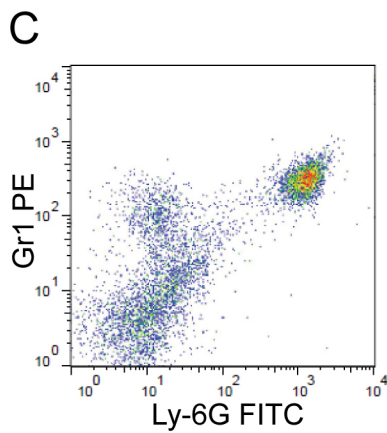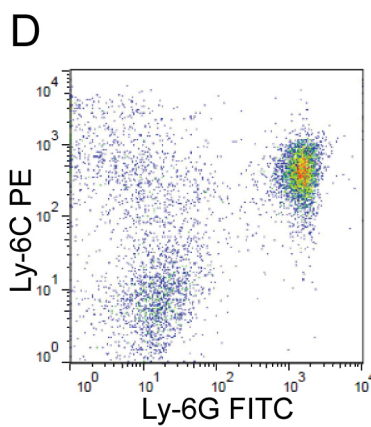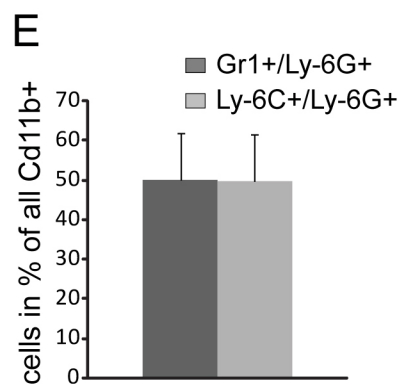

Supplement: Additional file 4 — Figure S4. Systemic enrichment of neutrophils in TgS100a8a9hepmice. (A) Relative expression of endogenous S100a9 transcripts was determined by qRT-PCR using cDNA from different tissues of Control (n=3) and TgS100a8a9hep (n=4) mice. Mean +SD, students t-test, *p≤0.05, **p≤0.01, n.s. not significant. (B) Absolute number of neutrophils in peripheral blood from Control and TgS100a8a9hep mice (n=4) as measured by means of blood counts. (C-E) Peripheral blood from Control mice (n=8) was subjected to tri-color staining using antibodies against Cd11b (APC), Ly-6G (FITC), and Gr1 (PE) or Ly-6C (PE), respectively. Representative images of either (C) Gr1/Ly-6G or (D) Ly-6C/Ly-6G populations are shown. (E) Graph shows the total percentage of Gr1+/Ly-6G+ cells and Ly-6C+/Ly-6G+ cells from all Cd11b+ cells in peripheral blood; +SD. [file 1478-811X-10-40-S4.pdf]

**A**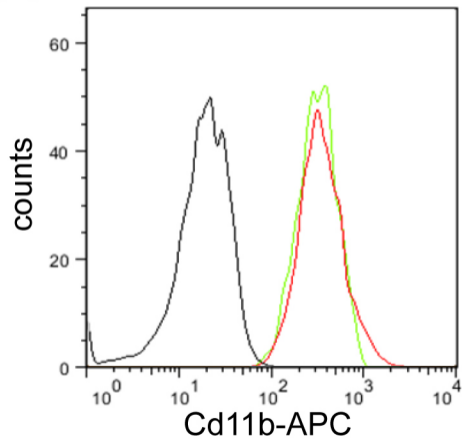**B**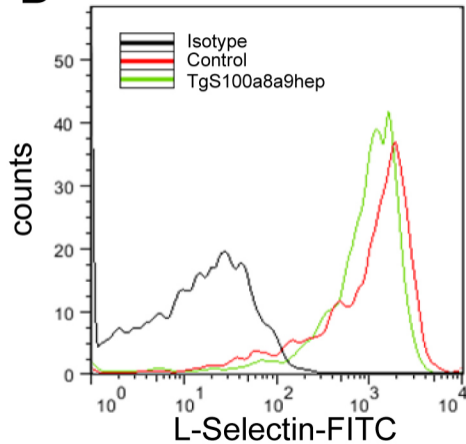

Supplement: Additional file 5 — Figure S5. Molecular markers of neutrophil activation in TgS100a8a9hepmice. Blood neutrophils (Gr1+/Ly6G+ or Cd11b+/Ly6G+) from Control and TgS100a8a9hep mice were stained with specific antibodies for Cd11b (APC) and L-Selectin (PE), or the respective isotype control antibody. Representative histograms of Cd11b (A) and L-Selectin (B) stainings are shown for Control (red) and TgS100a8a9hep (green) mice, including the staining with their respective isotype control (black). [file 1478-811X-10-40-S5.pdf]

**A**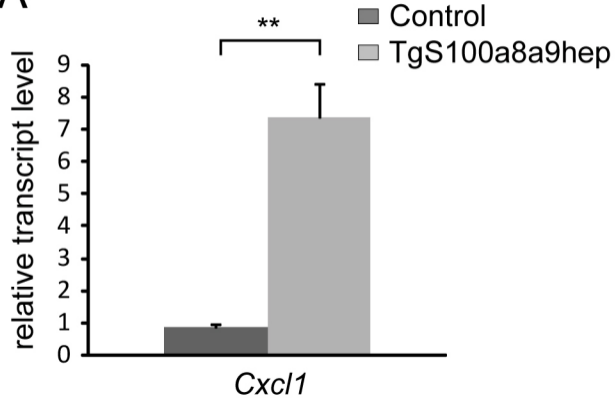**B**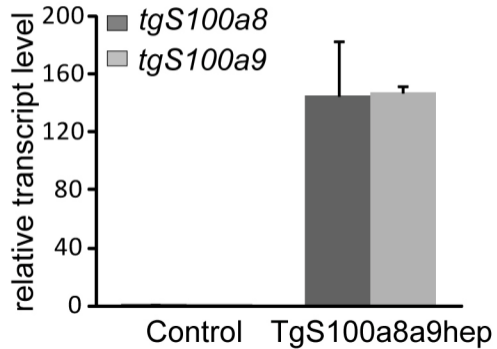

Supplement: Additional file 6 — Figure S6.Cxcl1 and tgS100a8/a9 expression in primary hepatocytes from Control and TgS100a8a9hepmice. (A) Relative Cxcl1 transcript levels was determined by qRT-PCR analysis with cDNA from cell suspension derived from collagenase perfused livers of Control and TgS100a8a9hep mice. (B) Primary hepatocytes were cultivated in vitro for 24 hours and relative levels of tgS100a8 and tgS100a9 transcripts were measured by qRT-PCR. Two biological replicates for each group were measured in triplicates and means are depicted +SD, students t-test, **p≤0.01. [file 1478-811X-10-40-S6.pdf]

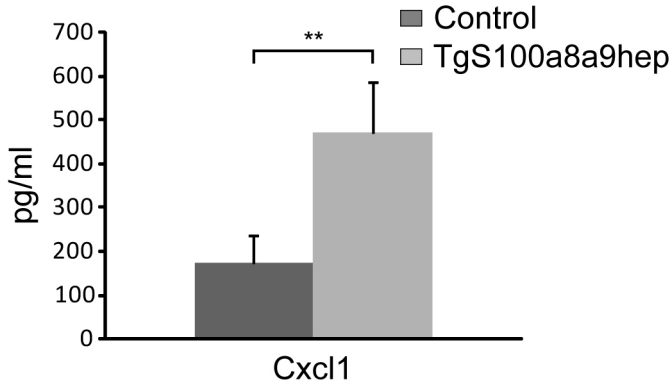

Supplement: Additional file 7 — Figure S7. Cxcl1 protein expression in liver lysates from Control and TgS100a8a9hepmice. Protein levels of Cxcl1 in liver lysate from Control and TgS100a8a9hep mice (n=4) were measured by ELISA. Mean +SD, students t-test, **p≤0.01. [file 1478-811X-10-40-S7.pdf]

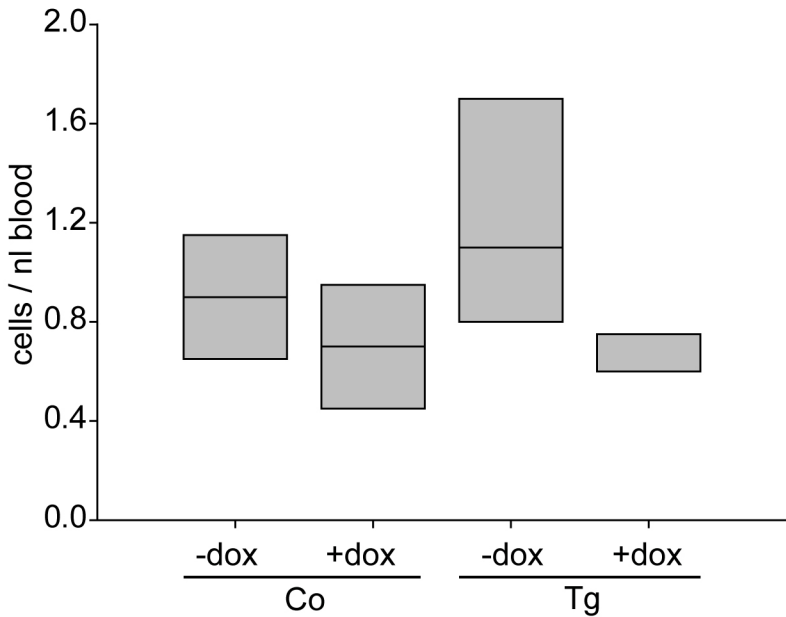

Supplement: Additional file 8 — Figure S8. Peripheral neutrophils in Control and TgS100a8a9hepmice treated with doxycycline. Control (Co) and TgS100a8a9hep (Tg) mice were treated with (Co +dox, Tg +dox) or without (Co -dox, Tg -dox) doxycycline (10 μg/ml) containing drinking water. Peripheral blood neutrophils were measured by means of blood counts and depicted as absolute numbers. Box-plot shows median, 25 % and 75 % quartile (light grey box). [file 1478-811X-10-40-S8.pdf]
