# Supplementary material for: Hepatocyte-specific S100a8 and S100a9 transgene expression in mice causes Cxcl1 induction and systemic neutrophil enrichment
Source: Cell Commun Signal. 2012 Dec 15;10:40. doi: 10.1186/1478-811X-10-40 (PMC3533587; doi:10.1186/1478-811X-10-40)
Supplement: Additional file 9 — Table S1. Antibodies used for IHC, Western blotting, and flow cytometry. [file 1478-811X-10-40-S9.pdf]

Table S1 - Antibodies used for IHC, Western blotting, and flow cytometry

Antibody	Cat.number	Company	Dilution
anti-Calgranulin A	sc-8113	Santa Cruz	1:200
anti-Calgranulin B	sc-8115	Santa Cruz	1:200
anti-Cd11b-APC	553312	BD Pharmingen	1:100
anti-Cd62L-PE	12-0621	eBioscience	1:3000
anti-cJun	#9165	Cell Signaling	1:1000
anti-ERK1/2	#4696	Cell Signaling	1:1000
anti-F4/80	MCA497R	Serotec	1:100
anti-Gr1 (RB6-8C5)	ab25377	Abcam	1:100
anti-Gr1-PE	12-5931	eBioscience	1:3000
anti-goat IgG, biotinylated	BA-9500	Vector Laboratories	1:500
anti-His	#493	MicroMol	1:1000
anti-HNE Michael Adducts	393207	Calbiochem	1:200
anti-rat IgG, biotinylated	BA-9500	Vector Laboratories	1:500
anti-Ly-6G-FITC	551460	BD Pharmingen	1:100
anti-Ly-6C-PE	12-5932	eBioscience	1:3000
anti-p38	#9218	Cell Signaling	1:1000
anti-p65	#3034	Cell Signaling	1:1000
anti-phospho c-Jun	#9261	Cell Signaling	1:1000
anti-phospho ERK1/2	#9101	Cell Signaling	1:1000
anti-phospho p38	#4511	Cell Signaling	1:1000
anti-phospho p65	#3036	Cell Signaling	1:1000
anti-phospho Stat 3	#9138	Cell Signaling	1:1000
anti-rabbit IgG, biotinylated	BA-1000	Vector Laboratories	1:500
anti-Stat3	#9132	Cell Signaling	1:1000
rat IgG2b κ Isotype APC	17-4031	eBioscience	1:100
rat IgG2b κ Isotype PE	12-4031	eBioscience	1:100
