# Supplementary material for: Hepatocyte-specific S100a8 and S100a9 transgene expression in mice causes Cxcl1 induction and systemic neutrophil enrichment
Source: Cell Commun Signal. 2012 Dec 15;10:40. doi: 10.1186/1478-811X-10-40 (PMC3533587; doi:10.1186/1478-811X-10-40)
Supplement: Additional file 10 — Table S2. Primer pairs used for qRT-PCR analysis. [file 1478-811X-10-40-S10.pdf]

Table S2 - Primer pairs used for qRT-PCR analysis

Primer	Sequence
endA8-F1	5`-CGT GAC AAT GCC GTC TGA-3`
endA8-R1	5`-AGG CCA GAA GCT CTG CTA-3`
endA9-F2	5`-AGC GCA GCA TAA CCA CCA TC-3`
endA9-R2	5`-GAC TTG GTT GGG CAG CAG TC-3`
mCsf1-F1	5`-GAG GTG TCA GAA CAC TGT AG-3`
mCsf1-R1	5`-CAA TCT GGC ATG AAG TCT CC-3`
mCsf3_F1	5`-CCA CCT TGG ACT TGC TTC AG-3`
mCsf3_R1	5`-TAC GAA ATG GCC AGG ACA CC-3`
mCxcl1-F1	5`-ACT GCA CCC AAA CCG AAG TC-3`
mCxcl1-R1	5`-TGT CAG AAG CCA GCG TTC AC-3`
mCxcl2-F3	5`-CAC CAA CCA CCA GGC TAC AG-3`
mCxcl2-R3	5`-GCT CCT CCT TTC CAG GTC AG-3`
mHprt1-F1	5`-CTG GTT AAG CAG TAC AGC CCC-3`
mHprt1-R1	5`-CAA AAG TCT GGG GAC GCA GC-3`
mS100a8-F3	5`- CGA AAT CAC CAT GCC CTC TAC AA-3`
mS100a8-R3	5`-ATG CCA CAC CCA CTT TTA TCA CC-3`
mS100a9-F1	5`-GGA GCG CAG CAT AAC CAC CAT C-3`
mS100a9-R1	5`-GCC ATC AGC ATC ATA CAC TCC TCA-3`
Myc	5`-CCT CTT CTG AGA TGA GTT TTT-3`
